# Association of physical activity with gut microbiome among low-income black American adults in the Southern Community Cohort Study

**DOI:** 10.1080/29933935.2025.2589861

**Published:** 2025-11-30

**Authors:** Jiajun Shi, Sang Minh Nguyen, Danxia Yu, Lei Wang, Lili Liu, Hui Cai, Jie Wu, Jirong Long, Qiuyin Cai, Martha J Shrubsole, Wei Zheng, Xiao-Ou Shu

**Affiliations:** a Division of Epidemiology, Department of Medicine, Vanderbilt Epidemiology Center, Vanderbilt-Ingram Cancer Center, Vanderbilt University Medical Center, Nashville, TN, USA; b Department of Medicine, School of Medicine, Vanderbilt University, Nashville, TN, USA

**Keywords:** Physical activity, exercise, gut microbiome, shotgun metagenomics, black American population

## Abstract

Physical activity (PA) has been suggested to influence the gut microbiome. We evaluated this association among low-income Black American adults. This study included 489 self-identified Black American participants from the Southern Community Cohort Study. PA data, including exercise/sport- and work/home-related moderate-vigorous PA (MVPA), was collected at cohort enrollment (2002−2009). Stool samples were collected between 2018 and 2021, and microbial composition was profiled using shotgun metagenomic sequencing. General linear regression models were employed to evaluate associations between PA and gut microbial *α*-diversity, abundance of individual species and metabolic pathways. Among all participants, MVPA measures were not associated with Shannon *α*-diversity (*p* > 0.05) and explained approximately 0.2−0.3% variation of Bray–Curtis dissimilarity. A total of 32 bacterial species, including seven *Bacteroides* species, two *Streptococcus* species, two *Prevotella* species, and nine microbial metabolic pathways, including D-fucofuranose biosynthesis, xyloglucan degradation, biosynthesis of L-citrulline, L-aspartate and L-asparagine biosynthesis, and urea cycle, were significantly associated with work/home-related and/or total MVPA (all false discovery rates < 0.10). In conclusion, MVPA, particularly from work and home activities, may modulate the composition and functionality of the gut microbiome among Black American adults.

## Article highlights

- Work/home-related moderate-vigorous physical activity (MVPA_w) and/or total MVPA (MVPA_a) comprising of MVPA_w and exercise/sport-related MVPA (MVPA_s) are associated with abundance of 32 gut bacterial species in Black American adults.
- MVPA_w and/or MVPA_a are associated with nine gut metabolic pathways.

## Introduction

Regular physical activity (PA) plays a crucial role in human health via preventing and aiding recovery from numerous cardiometabolic and inflammatory diseases.^1^^‐^^3^ PA has been shown to improve lipid profiles, blood pressure, glucose intolerance, thrombosis, self–satisfaction, and immune functions.^4^^‐^^7^ Emerging evidence suggests that the health benefits of PA may, in part, be mediated through its influence on the gut microbiome.^8^^‐^^10^

However, previous human studies examining the relationship between PA and gut microbiome have primarily focused on structured exercise training or sports participation, often with relatively small sample sizes (*n* < 100) and results that are not entirely consistent.^8^^‐^^14^ The few population–based observational studies that have explored PA–gut microbiome associations^15^^‐^^19^ were limited by their focus on narrowly defined PA traits (e.g., leisure–time exercise or professional sports training), inclusion of primarily Europeans or East Asians, or insufficient adjustment for key confounders, such as dietary factors and medication use. Work–or housework–related PA, which constitute a substantial proportion of total PA in the general population, have seldom been included in gut microbiome association investigations.^15^^‐^^17^^,^^20^

In the present study, we hypothesised that in older, low socioeconomic populations work/home–related moderate–to–vigorous physical activity (MVPA_w) contributes more to total MVPA (MVPA_a) and is more likely to be associated with gut microbiota than exercise/sport related MVPA(MVPA–s). We evaluated the associations of multiple PA measures with gut microbiome in low–income Black American adults enroled in the Southern Community Cohort Study (SCCS). Specifically, we examined the relationships of MVPA_w, MVPA_s, MVPA_a, and sedentary behaviour at cohort enrolment with gut microbial diversity, species abundance, and microbiome–informed metabolic pathways.

## Materials and methods

### Study participants and data collection

The SCCS is an ongoing prospective cohort study designed to investigate risk factors of chronic diseases in a predominantly low–income population, as described in detail elsewhere.^21^^,^^22^ Briefly, between March 2002 and September 2009, approximately 86,000 adults (aged 40−79 years) were recruited from 12 Southeastern U.S. states, two–thirds of whom self–identified as Black American. Approximately 86% of participants were recruited from community health centres (CHCs), institutions providing basic health care and preventative services in underserved areas, so that the cohort encompassed a substantial number of individuals characterised by low–income, low–education, and lack of insurance. The remaining 14% of cohort members were recruited through mail–based general population sampling. The SCCS was reviewed and approved by the institutional review boards at Vanderbilt University and Meharry Medical College. Written informed consent was obtained from all study participants.

For the present study, we analysed a sub–cohort of SCCS participants who provided stool samples during follow–up, as described below.

### Stool sample collection, microbial DNA extraction, and metagenomic sequencing

Stool samples were collected from a subset of the SCCS participants between 2018 and 2021, 9.0 to 18.1 (mean 13.8) years later from cohort enrolment, using faecal occult blood test (FOBT) cards following a standard protocol.^23^ Participants were mailed a FOBT stool collection kit, asked to collect two small pieces of stool, sign a consent form, complete a concise survey, and ship back the above items within 24 hours with prepared postage to the study’s laboratory. The stool samples were stored at −80 °C until further use.

DNA extraction from stool samples of 594 Black American participants was performed using the DNeasy PowerSoil Kit (Qiagen, Carlsbad, CA, USA) following the manufacturer's protocols. Subsequently, sequencing libraries for whole–genome shotgun metagenomic sequencing were constructed from the DNA samples using the DNBSEQ short–read library preparation kit (MGI Tech, Shenzhen, China). Sequencing was performed in paired–end 150 bp mode on the MGISEQ−2000 platform at BGI Americas (Cambridge, MA, USA). DNA extraction, library preparation, and sequencing of all stool samples were done in a single batch.

Sequencing reads per sample ranged between 12,185,928 and 18,874,961, with a mean of 17,470,242. Sequencing reads were subjected to quality trimming via Trimmomatic v0.39,^24^ and Bowtie2 v2.3.0^25^ was used to remove human reads. We used Kraken v2.1.1^26^ to classify microbial species, with 286,997 prokaryotic genomes and 4,644 representative species from the Unified Human Gastrointestinal Genome (UHGG) collection version 1.0^27^ (http://ftp.ebi.ac.uk/pub/databases/metagenomics/mgnify_genomes/human-gut/v1.0/) as the reference set. Absolute and relative abundance of microbial species for each sample were estimated using Bracken^28^ (v2.6, default parameters). HUMAnN 3.0^29^ was utilised to perform functional profiling of the gut microbiome using clean reads and the UniRef90 comprehensive protein database^30^ as reference. We estimated the relative abundance of gut microbial metabolic pathways using the MetaCyc database (version 24.0) as the reference.^31^

For the current analysis, we excluded participants who lacked PA data (*n* = 30), had bowel preparation (*n* = 33), experienced diarrhoea (*n* = 3), used antibiotics (*n* = 5) during the past two months or used probiotic supplements (*n* = 36) in the past week prior to stool sample collection, or reported having inflammatory bowel disease at baseline (*n* = 2). A total of 489 participants were included in the final analysis.

### Measurement of physical activity and sedentary behaviours

Usual occupational, household, and leisure time PA and sedentary behaviour were assessed using a validated, SCCS–specific Physical Activity Questionnaire (PAQ)^32^ at the time of the cohort enrolment. PAQ and detailed descriptions of physical activity and derived variables for the SCCS are included in the baseline questionnaire and the updated codebook in 2017 at https://www.southerncommunitystudy.org/questionnaires.html. Briefly, participants were queried about the duration typically spent performing light, moderate, and vigorous/strenuous activities at work and/or at home, as well as moderate and vigorous exercise or sports in leisure time. Examples of light work were given to participants, including standing at work, shopping, cooking, and child or elderly care. Moderate work examples included shop work, cleaning house, gardening, mowing lawns and home repair. Examples of strenuous work included loading or unloading trucks, construction, farming or other hard labour. Moderate sports encompassed activities such as bowling, dancing and golfing, while vigorous sports included jogging, aerobics, tennis, swimming and weightlifting. Time allocation for work and home activities was assessed separately for weekdays and weekends and then combined using weighted averages with assigned weights of 5/7 and 2/7, respectively (see physical activity scoring in the appendix of the SCCS code book), while exercise and sports participation were assessed for a typical week. Total PA was estimated as the sum of home and work activity as well as exercise and sports participation. Regarding sedentary behaviours, participants were asked questions about the amount of time per day typically spent sitting in a car or bus, at work, viewing television or movies, and other activities that involve sitting, such as sitting at meals, talking on the phone, reading, playing games, or sewing.

For analysis, duration and type of active activities were used to estimate physical activity energy expenditure in metabolic equivalent (MET) hours per day using common MET values for the specific activities assessed using the Compendium of Physical Activities.^33^ One MET–hour/day of energy expenditure can be achieved by participating in 0.5 hours of any light activity (2 METs per hour), 0.25 hours of any moderate intensity activity (4 METs per hour), or 0.125 hours of any vigorous activity (8 METs per hour). Given that over 70% of the participants reported no moderate or vigorous exercise/sports (MVPA_s), we also derived moderate and vigorous home/work activities (MVPA_w) and all/total moderate and vigorous activity (MVPA_a), and treated them as continuous exposures in this study, which were denoted in MET–hours per week (MET–h/w). MVPA was stratified into binary variables by sex–specific medians, resulting in MVPA_sC (any vs. none), MVPA_wC (high vs. low), and MVPA–aC (high vs. low). Due to the high correlation between MVPA_a and total PA (Spearman correlation rho = 0.92) and given that light PA was not shown previously to be associated with gut microbiota, we did not include these two PA measures in our study. In addition, the duration of sedentary behaviours was summarised and expressed in hours per day (TotalSit_hrs). Full names and definition of PA measures/traits and sitting behaviours for this study are included in Table S1.

### Assessment of covariates

Baseline information included as covariates in our study were demographic factors (age, sex, race, educational level, and annual household income), body mass index (BMI, calculated by weight in kilograms divided by the square of height in metres), recruitment source (CHC or general population), pack–years of smoking, alcohol intake (drinks per day), total energy intake, healthy eating index (HEI), and history of cancer, cardiovascular disease (CVD), diabetes, and hypertension. The dietary intake was assessed using an 89-item food frequency questionnaire (FFQ) developed specifically for the typical diet in the southeastern United States and validated with respect to a number of nutrients.^34^^,^^35^ The Healthy Eating Index−2010 (HEI−10) was calculated for participants who completed at least 79 of the 89 FFQ questions and had energy intake within the range of 600 to 8,000 kcal/day, and with a score ranging between 0 and 100, as described in detail elsewhere.^36^^‐^^38^

### Statistical analysis

Study participant characteristics were compared between MVPA_sC and MVPA_wC groups using nonparametric Wilcoxon rank sum tests for continuous variables and chi–square tests for categorical variables. To estimate the richness/evenness and diversity of the gut microbiota, the species level absolute abundance data were rarefied to the minimal sequencing reads (*n* = 12,185,928). Species level alpha and beta diversity were measured by the Shannon index and Bray–Curtis distance matrix, respectively, derived by the R *vegan* package (v2.5−7).^39^ The associations of Shannon diversity with categorical MVPA (i.e., MVPA_sC, MVPA_wC, and MVPA_aC) were examined via general linear regression models. The proportion of variation in the Bray–Curtis dissimilarity explained by categorical MVPA and TotalSit–hrs was estimated using permutational multivariate analysis of variance (PERMANOVA) using the *adonis2* function included in the R package *vegan*.^39^ Bray–Curtis matrix difference of any vs. none MVPA_s participants, or high vs. low MVPA_w participants was visualised by Principal Coordinates Analysis (PCoA) and compared using a microbiome regression–based kernel association test (MiRKAT).^40^

Associations of continuous and categorical MVPA measures and TotalSit–hrs with individual gut bacterial species or microbiome–inferred metabolic pathways were estimated using general linear regression models. Analysis was restricted to all species and metabolic pathways that had a median relative abundance (RA) of >0.001% and a prevalence (carrier frequency) of >20% in our participants. The absolute abundance (i.e., sequencing counts) for each of the 1,888 selected species included in the analyses were normalised using the centred log–ratio transformation after adding 1 as a pseudo–count.^41^ RA of metabolic pathways was normalised using a 2-fold arcsine square root transformation.

For all the above association analyses with MVPA measures using PERMANOVA, MiRKAT, or general linear regression models, potential confounders adjusted included enrolment age, sex, time interval between cohort enrolment and stool sample collection, education, annual household income, baseline BMI, tobacco smoking, alcohol drinking, total energy intake, HEI−10, daily sitting hours, and history of selected chronic diseases at baseline including cancer, cardiovascular disease, diabetes, and hypertension. For TotalSit_hrs associations, total PA was additionally adjusted. In sensitivity analysis, bowel movement frequency or stool appearance/type during stool sample collection were separately added to the regression models. Missing values for confounders/covariates (typically for <0.2−6.0% of participants) were imputed with sex–specific medians or mode values of our study population. We also conducted stratified analyses for PA measures associated with significant microbial species and metabolic pathways by age at stool sample collection (median), sex, BMI (obese or not), annual household income (< or ≥ $15,000), smoking status (ever or never), and drinking alcohol (ever or never). FDR was used to account for multiple testing; an FDR *q*-value < 0.10 was considered statistically significant. All analyses were performed using SAS Enterprise Guide 7.1 (SAS Institute Inc.) or R version 4.2.1 unless otherwise indicated.

## Results

### Characteristics of the study subjects

Among 489 participants, the mean age at stool sample collection was 66.4 years [standard deviation (sd), 7.8 years; range, 50−90 years], and 138 (28.2%) were males. Table 1 presents the distribution of selected characteristics for study participants by categorical exercise/sport–related or work/home–related MVPA. Compared with non–exercisers, exercisers were more likely to have a college education and higher household income, total caloric intake, and HEI−10 scores, but fewer comorbidities such as obesity, diabetes, and hypertension. Compared with low MVPA_w participants, high MVPA_w participants were younger and less likely to have a college education and a comorbidity of cancer, but more likely to have lower HEI scores and higher total caloric intake (all *p* < 0.05).

**Table 1.** Selected characteristics of participants from the Southern Community Cohort Study.

	Sport–related moderate to vigorous physical activity (MVPA_sC)			Work–related moderate to vigorous physical activity (MVPA_wC)		
Characteristics	None (*n* = 327)	Any (*n* = 162)	*P* ^‡^	Low MET–h/w (females < 56, males < 68; *n* = 220)	High MET–h/w (females ≥ 56, males ≥ 68; *n* = 269)	*P* ^‡^
Age at baseline, years, median (IQR)	53 (47−59)	52 (47−57)	0.201	54 (49−59)	51 (46−57)	0.010
Age at stool sample collection, years, median (IQR)	66 (61−72)	66 (60−70)	0.294	67 (62−72)	65 (60−70)	0.024
Males, *n* (%)	89 (27.22)	49 (30.25)	0.553	70 (31.82)	68 (25.28)	0.134
Education, *n* (%)			<0.001			0.003
<High school	69 (21.10)	17 (10.49)		39 (17.73)	47 (17.47)	
High school	115 (35.17)	52 (32.10)		65 (29.55)	102 (37.92)	
Some college	105 (32.11)	52 (32.10)		66 (30.00)	91 (33.83)	
≥College degree	38 (11.62)	41 (25.31)		50 (22.73)	29 (10.78)	
Annual household income, *n* (%)			<0.001			0.119
<$15,000	148 (45.26)	59 (36.42)		94 (42.73)	113 (42.01)	
$15,000-<$25,000	95 (29.05)	37 (22.84)		53 (24.09)	79 (29.37)	
$25,000-<$50,000	60 (18.35)	28 (17.28)		37 (16.82)	51 (18.96)	
≥$50,000	24 (7.34)	38 (23.46)		36 (16.36)	26 (9.67)	
BMI, kg/m^2^, *n* (%)			0.028			0.640
<25	61 (18.65)	28 (17.28)		37 (16.82)	52 (19.33)	
25-<30	83 (25.38)	60 (37.04)		66 (30.00)	77 (28.62)	
30-<35	94 (28.75)	45 (27.78)		59 (26.82)	80 (29.74)	
≥35	89 (27.22)	29 (17.90)		58 (26.36)	60 (22.30)	
Pack–years of smoking, *n*, median (IQR)^†^	13.0 (6.0, 24.0)	12.0 (6.5, 19.5)	0.477	13.0 (5.2, 24.0)	12.5 (7.0, 23.0)	0.861
Alcohol drinks per day, *n*, median (IQR)	0 (0−0.20)	0 (0−0.50)	0.087	0.0 (0.0, 0.4)	0.0 (0.0, 0.2)	0.714
Total energy intake, kcal/day, median (IQR)	2037 (1474−2791)	2323 (1578−3206)	0.012	1944 (1469, 2666)	2209 (1570, 3123)	0.027
Healthy eating index, median (IQR)	59.3 (49.0−68.0)	62.8 (55.0−72.1)	<0.001	62.8 (53.7, 71.4)	59.4 (51.1, 67.5)	0.017
History of cancer, *n* (%)	19 (5.81)	15 (9.26)	0.222	23 (10.45)	11 (4.09)	0.010
History of cardiovascular disease, *n* (%)	24 (7.34)	8 (4.94)	0.414	17 (7.73)	15 (5.58)	0.439
History of diabetes, *n* (%)	62 (18.96)	18 (11.11)	0.038	42 (19.09)	38 (14.13)	0.176
History of hypertension, *n* (%)	201 (61.47)	76 (46.91)	0.003	125 (56.82)	152 (56.51)	0.999
Total physical activity, MET–h/d, median (IQR)	15.1 (8.1, 26.3)	21.7 (13.7, 32.5)	<0.001	9.2 (5.2, 13.7)	26.2 (19.1, 37.2)	<0.001
Total sitting hours/d, *n*, median (IQR)	8.6 (6.0, 12.5)	9.0 (6.5, 13.0)	0.434	8.5 (6.0, 12.7)	9.0 (6.5, 12.6)	0.220

^†^ Among ever smokers.

^‡^ *P* values based on nonparametric Wilcoxon rank sum tests for continuous variables and chi–square tests for categorical variables.

### Alpha and beta diversity of gut bacteria

**Figure 1.**
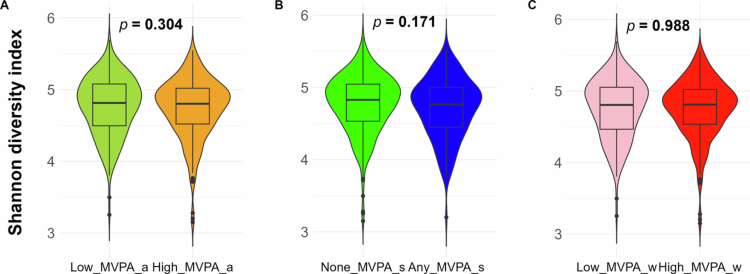
Difference in Shannon diversity between moderate to vigorous physical activity (MVPA) groups of the participants (left: combined exercise/sport and work/home [MVPA_a]; middle: exercise/sport [MVPA_s]; right: work/home [MVPA_w]).

**Figure 2.**
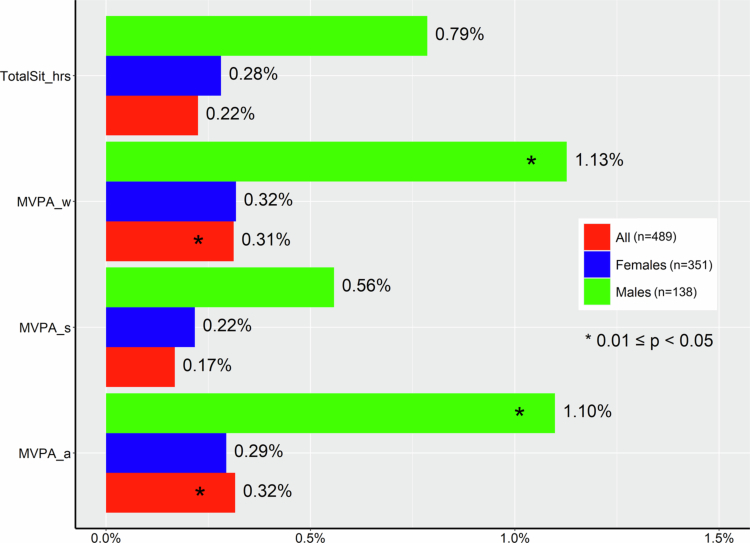
Proportion of variation (R^2^) in species–level Bray–Curtis dissimilarity explained by daily sitting hours (TotalSit_hrs) and moderate to vigorous physical activity (MVPA) measures, including those related to exercise/sport (MVPA_s), work/home (MVPA_w), and all (MVPA_a). Results were derived from permutational multivariate analysis of variance on the Bray–Curtis dissimilarities among males, females, and all 489 participants.

**Figure 3.**
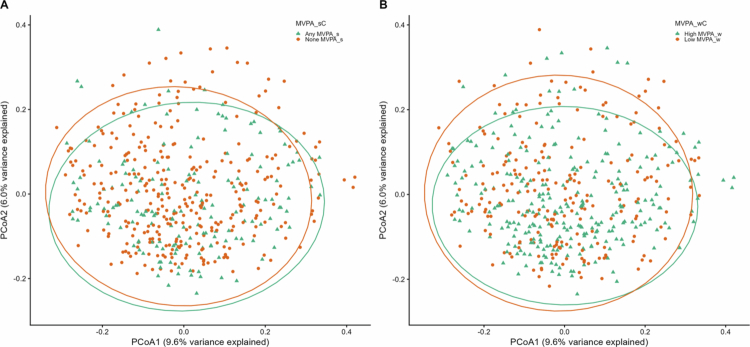
PCoA plot of microbial Bray–Curtis dissimilarity between MVPA groups. (A) Any vs. none exercise/sport (MVPA_s). (B) High vs. low work/home physical activity (MVPA_w).

Mean (sd) of *α*-diversity Shannon index were 4.74 (0.41) and 4.77 (0.40) for participants with high and low levels of total MVPA, 4.71 (0.41) and 4.78 (0.40) for participants engaged with any or no sport/exercise, and 4.76 (0.40) and 4.75 (0.42) for participants with high and low levels of work/home–related MVPA, respectively. None of the three categorical MVPA measures were associated with Shannon diversity (Figure 1, all *p* > 0.05). However, when compared between MVPA groups, males that engaged in any exercise/sport (MVPA_sC) exhibited significantly lower Shannon index than those that did not engage any exercise/sport [mean (sd): 4.69 (0.45) vs. 4.91 (0.36), *p* = 0.006, Figure S1]. Continuous MVPA measures and daily sitting hours explained less than 0.32%, 0.32%, and 1.13% variation in Bray–Curtis dissimilarity among all participants, females, and males, respectively (Figure 2**)**. We did not find any significant difference between MVPA subgroups in Bray–Curtis dissimilarity, with an omnibus MiRKAT *p* = 0.660 for any vs. none MVPA_s, and *p* = 0.994 for high vs. low MVPA_w, and indicated by PCoA plots (Figure 3). In addition, PERMANOVA identified significant effects of several potential confounders, including age (R^2^ = 0.33%, *p* = 0.011), sex (R^2^ = 0.53%, *p* = 0.001), and history of diabetes (R^2^ = 0.32%, *p* = 0.024) and hypertension (R^2^ = 0.29%, *p* = 0.045) on microbiome composition (Figure S2).

### Associations of PA with individual microbial species and metabolic pathways

**Figure 4.**
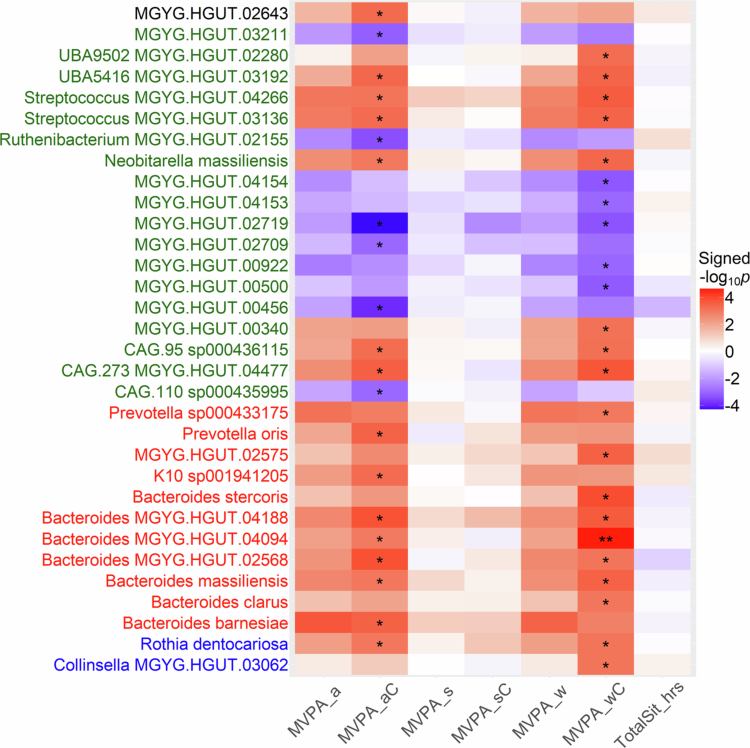
Associations between physical activity measures and gut microbial species in the Southern Community Cohort Study. The significant heatmap cells are noted by FDR *q* values (*0.05 ≤ *q* < 0.10; **q < 0.05) and the directions of associations are represented by colours, with red signifying positive beta coefficients and blue signifying negative beta coefficients from linear regression models. Phyla for species are indicated by text colours: blue for *Actinobacteriota*, red for *Bacteroidota*, green for *Firmicutes*, and black for *Proteobacteria*.

We examined 1,888 species, which were taxonomically classified into 581 genera, 115 families, 49 orders, 23 classes, and 17 phyla. Among these, 32 species (see full names from UHGG in the top of Table S1) were significantly associated with MVPA measures at an FDR *q*-value < 0.10 among all participants (Figure 4 and Table S2). Notably, MVPA_wC and/or MVPA_aC were positively associated with the abundance of seven *Bacteroides* species, including *B. barnesiae*, *B. clarus*, B*. massiliensis*, *B. stercoris*, and three unclassified species (beta range: 0.620 to 1.114; 0.045 < *q* < 0.098). Both MVPA_wC and MVPA_aC showed positive associations with two unclassified *Streptococcus* species, including *S. MGYG.HGUT.03136* and *S. MGYG.HGUT.04266* (beta range: 0.879 to 1.070). *Prevotella oris* was positively associated with a higher level of total MVPA (beta = 0.543, *q* = 0.082) while *P. sp000433175* was associated with higher MVPA_w (beta = 0.972, *q* = 0.085). We identified 10 unclassified species negatively associated with high level of MVPA_w and/or MVPA_a (beta range: −1.233 to −0.462). TotalSit_hrs was not significantly associated with the abundance of any species evaluated. Additional adjustment for bowel movement frequency or stool appearance/type did not materially alter the association effect sizes for these species (Figures S3 and S4).

**Figure 5.**
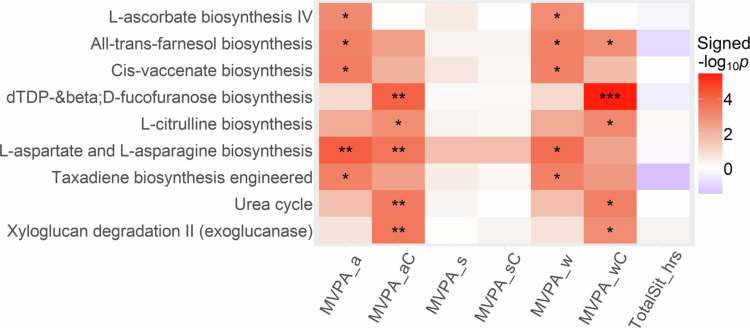
Associations between physical activity measures and gut microbial metabolic pathways in the Southern Community Cohort Study. The significant heatmap cells are noted by FDR *q* values (*0.05 ≤ *q* < 0.10; **0.01 ≤ *q* < 0.05; ***q < 0.01) and the directions of associations are represented by colours, with red signifying positive beta coefficients and blue signifying negative beta coefficients from linear regression models.

Among 428 microbial metabolic pathways examined, nine (full names at the bottom of Table S1) were significantly associated with one or more work/home–related or total MVPA measures in all participants (Figure 5 and Table S3). The prominent pathways included glucose metabolism (i.e., D–fucofuranose biosynthesis and xyloglucan degradation), amino acid metabolism (i.e., L–aspartate, L–asparagine, and L–citrulline), alcohol metabolism (i.e., all–trans–farnesol), taxadiene biosynthesis, L–ascorbate biosynthesis, and urea cycle. Notably, all the pathway associations with MVPA measures were positive. No significant associations were observed between TotalSit_hrs and relative abundance of any of the metabolic pathways. Similarly, further adjustment for bowel movement frequency or stool appearance/type did not meaningfully change these pathway–specific associations (Figures S5 and S6).

Our stratified analyses revealed different effect modifications of smoking status on the associations of total and sport–related MVPA with gut microbial species (Table S4, FDR *q* < 0.10 for interactions). MVPA_aC was significantly associated with a lower abundance of the unclassified species *MGYG.HGUT.00922* within order *Christensenellales* among ever smokers (beta = −1.814, *p* = 1.64 × 10^−5^) but not among never smokers (beta = 0.321, *p* = 0.471). Opposite associations between MVPA_sC and *CAG.95 sp000436115* (in family *Lachnospiraceae*) were also observed in subgroups by smoking status (beta = 0.634 in ever smokers; beta = −0.439 in never smokers). Similarly, positive associations of both continuous and categorical sport/exercise–related MVPA measures with the L–ascorbate biosynthesis IV pathway were observed among participants with an annual household income of <$15,000, while negative associations were observed among participants with higher income (Table S5).

## Discussion

In this study of 489 low–income Black American adults, we observed that approximately 0.3% variation of the Bray–Curtis dissimilarity was explained by either work/home–related MVPA or overall MVPA. A total of 32 microbial species, including seven *Bacteroides* species/subspecies, two *Streptococcus* species, and two *Prevotella* species, were significantly associated with categorical work/home–related and/or total MVPA measures after multiple comparison corrections. MVPA measures were also associated with nine gut microbial metabolic pathways. In addition, smoking status and household income may modify the associations between MVPA and two unclassified microbial species and one metabolic pathway, respectively.

Gut microbial community changes induced by PA, particularly regular exercise or sports training, encompass intra–individual *α*-diversity and inter–individual *β*-diversity.^8^^‐^^14^^,^^17^ Exercise has been hypothesised to increase gut microbial *α*-diversity, thus enhancing microbial stability, metabolic capacity, and the ability to defend exotic invading pathogens.^10^^,^^12^ Several cross–sectional or observational studies have reported significant associations with PA, but rarely for the same PA measures or from general populations.^17^^,^^19^^,^^42^^‐^^46^ In our study, MVPA measures were not associated with *α*-diversity, consistent with most previous studies.^8^^,^^47^^‐^^49^ However, it remains unclear why males who engaged in exercise/sport–related MVPA exhibited significantly lower Shannon diversity compared to those who did not engage in this form of PA. We further investigated the relationship between different PA measures and gut community composition. While prior population–based studies have reported that PA accounts for approximately 1% of the *β*-diversity variation,^15^^,^^16^ our findings suggest that both work/home–related and total MVPA explained only 0.3% of the gut microbial variation in Bray–Curtis diversity among predominantly low–income Black American adults. Consistent with our previous study among elderly urban Chinese individuals,^18^ exercise/sport–related PA contributed to less than 0.2% variation in gut microbial *β*-diversity among elderly Black American individuals. When comparing *β*-diversity variations explained by different MVPA measures, work/home–related PA (i.e., MVPA_w) contributed more than exercise/sport–related PA (Figure 2). Given that MVPA_w constitutes a substantial proportion of total MVPA in SCCS participants and similar low–income adult populations, future studies on PA–gut microbiome association should consider this type of PA.

PA may impact individual microbial taxa in the gut.^8,10,11,47^^‐^^49^ However, due to variations in PA type, intensity, and duration, as well as study design, participant characteristics, and adjustment for confounders (e.g., age, sex, dietary measures, other lifestyles, and clinical factors), few gut bacterial taxa have been consistently associated with PA. In a population study, genus *Veillonella* remained positively associated with vigorous PA after adjusting for multiple confounding factors.^17^ This genus has also been enriched in marathon runners^50^ and in diabetic and prediabetic individuals undergoing moderate intensity continuous training.^51^ However, in our analysis, *Veillonella* was only marginally associated with vigorous PA (beta = 0.023, se = 0.012, *p* = 0.056). Similarly, while increased *Prevotella* abundance has been reported in athletes,^42^^,^^45^^,^^52^ we did not observe a significant association between this genus and vigorous PA (beta = 0.011, se = 0.006, *p* = 0.076). However, *Prevotella oris* was positively associated with MVPA_aC and *P. sp000433175* with MVPA_wC (Figure 4 and Table S2). Further research is warranted to elucidate whether an elevated *Prevotella* abundance is correlated with specific types of PA/exercise (e.g., sports training) and whether such alterations confer human health benefits.^53^

Interestingly, prior studies reported negative associations of the genus *Bacteroides* with walking or biking (FDR < 0.05), nominal negative associations with vigorous activity, and positive associations with moderate activity.^17^ In our metagenomic analysis, 81 *Bacteroides* species (relative abundance > 0.001%, prevalence > 20%) were detected across 489 participants, with seven significantly and positively associated with work/home–related MVPA (Figure 4). These taxa may represent a co–occurring sub–community,^54^^,^^55^ reflecting functional adaptations to this activity domain.

Our analysis of microbial metabolic pathways revealed potential biological functions underlying the PA–gut microbiota associations. For example, MVPA was also positively correlated with the urea cycle pathway inferred from the gut microbiome.^56^ The enrichment of the xyloglucan degradation pathway may indicate enhanced energy harvest from dietary fibre, a healthy behaviour that commonly accompanies physical acidity, through increased activity of gut microbial taxa.^57^ Moreover, work/home–related MVPA was associated with increased microbial L–citrulline biosynthesis, a pathway implicated in anti–inflammatory, antioxidant, and immunomodulatory effects in the gut.^58^ Nevertheless, whether these pathway associations are specific to work/home–related MVPA and the exact mechanisms by which they influence health in older adults remain largely unclear.

A key strength of this study is its prospective investigation of the long–term effects of both exercise/sport–and work/home–related MVPA, as well as daily sitting hours, on the gut microbiome in a large low–income Black American population. Notably, this is among the largest gut microbiome studies to examine physical activity related to work, occupation, and home maintenance.^20^^,^^59^ Our findings underscore the importance of considering this activity domain in gut microbiome research. To minimise potential confounding, our association analyses adjusted for a wide range of covariates. By leveraging shotgun metagenomic sequencing data, we comprehensively examined the PA–associated microbial communities, individual microbial species, and metabolic pathways, allowing us to explore potential biological mechanisms underlying PA–gut microbiota associations.

However, several limitations of this study should be acknowledged. First, measurement errors may exist due to self–reported physical activity, diet/lifestyle, and other health–related characteristics. The use of accelerometer–based physical activity measures could help reduce measurement errors. Second, the one–time PA assessment precluded evaluation of the effects the changes of PA overtime had on the gut microbiota. Over the mean 13.8-year interval (sd, 1.9 years) between cohort enrolment and stool sample collection, changes in the physical activity levels and other potential confounding factors such as tobacco smoking behaviour, diet, and health status between baseline and stool sample collection may have influenced our findings. Additionally, due to the lack of stool samples at baseline and PA data at the time of stool collection, we were unable to assess the trajectory of these associations. Third, while most significant associations in the present study were observed with work/home–related MVPA, it remains unclear to what extent these findings apply to other PA types (e.g., structured exercise), which may be more modifiable for prospective public health interventions. Fourth, while several previous studies also included participants of African–ancestry,^15^^,^^17^^,^^19^^,^^44^ the number of Black subjects was small (*n* < 300) and race–specific PA–associated results were unavailable. Our study though, has the largest number of Black participants of any study to date, and most were from low–income backgrounds. The findings of our study may not be generalisable to the Black population or other populations. Finally, although we identified 32 gut microbial species and nine inferred metabolic pathways associated with MVPA, few have been previously linked to underlying mechanisms such as altered production of bile acids, short–chain fatty acids, lipopolysaccharide, and immunoglobulin A in the context of PA/exercise–modulated gut microbiota.^8^^,^^13^^,^^56^ Further studies integrating metabolomics, lipidomics, intestinal transit time, and health or disease outcomes are needed to clarify the molecular and physiological mechanisms underlying our findings.

## Conclusion

In summary, this metagenomic study among older, low–income Black Americans identified significant associations of 32 microbial species and nine metabolic pathways with moderate–vigorous physical activities, mostly ascribed to those work/home–related. Further studies with larger sample sizes, across various populations, and using more comprehensive multi–omics approaches are warranted to further explore the associations between both sport/exercise–and work/home–related physical activities and gut microbial composition, as well as the underlying biological mechanisms.

## Supplementary Material

Supplementary Material PA and gut microbiome_Supplementary_material

## Data Availability

The deidentified raw metagenomic sequencing data will be deposited in the database of Genotypes and Phenotypes (dbGaP). Baseline Questionnaire and Stool Questionnaire for the SCCS are publicly available at https://ors.southerncommunitystudy.org/Home/Documentation. Other datasets and statistical codes used in the present study can be requested through the SCCS Online request System (https://ors.southerncommunitystudy.org).
