# Supplementary material for: Association of physical activity with gut microbiome among low-income black American adults in the Southern Community Cohort Study
Source: Gut Microbes Rep. 2025 Nov 30;2(1):2589861. doi: 10.1080/29933935.2025.2589861 (PMC12940208; doi:10.1080/29933935.2025.2589861)
Supplement: Supplementary Material — PA and gut microbiome_Supplementary_material [file KGMR_A_2589861_SM5076.docx]

**Supplement to “Association of physical activity with gut microbiome among predominantly low-income Black American adults in the Southern Community Cohort Study”**

**Contents of Supplemental Material**

**Figure S1** Difference in Shannon diversity between moderate to vigorous physical activity (MVPA) groups of the participants, by sex and PA types

**Figure S2** Proportion of variation in species-level Bray–Curtis dissimilarity explained by potential confounders in permutational multivariate analysis of variance wherein total moderate to vigorous physical activity served as the main exposure

**Figure S3** Associations between physical activity measures and gut microbial species in the Southern Community Cohort Study, with additional adjustment for **bowel movement frequency**

**Figure S4** Associations between physical activity measures and gut microbial species in the Southern Community Cohort Study, with additional adjustment for **stool appearance/type**

**Figure S5** Associations between physical activity measures and gut microbial metabolic pathways in the Southern Community Cohort Study, with additional adjustment for **bowel movement frequency**

**Figure S6** Associations between physical activity measures and gut microbial metabolic pathways in the Southern Community Cohort Study, with additional adjustment for **stool appearance/type**

**Table S1** Abbreviations of exposures, microbial species, and metabolic pathways in this study

**Table S2** Microbial species associated with physical activity measures in all selected SCCS participants

**Table S3** Microbial MetaCyC pathways associated with physical activity measures in all selected SCCS participants

**Table S4** Significant interactions in moderate to vigorous physical activity and gut microbial species associations

**Table S5** Significant interactions in physical activity measures and metabolic pathway associations

**Figure S1** Difference in Shannon diversity between moderate to vigorous physical activity (MVPA) groups of the participants, by sex (top: males; bottom: females) and PA types (left: combined exercise/sport and work/home [MVPA_a]; middle: exercise/sport [MVPA_s]; right: work/home [MVPA_w]).


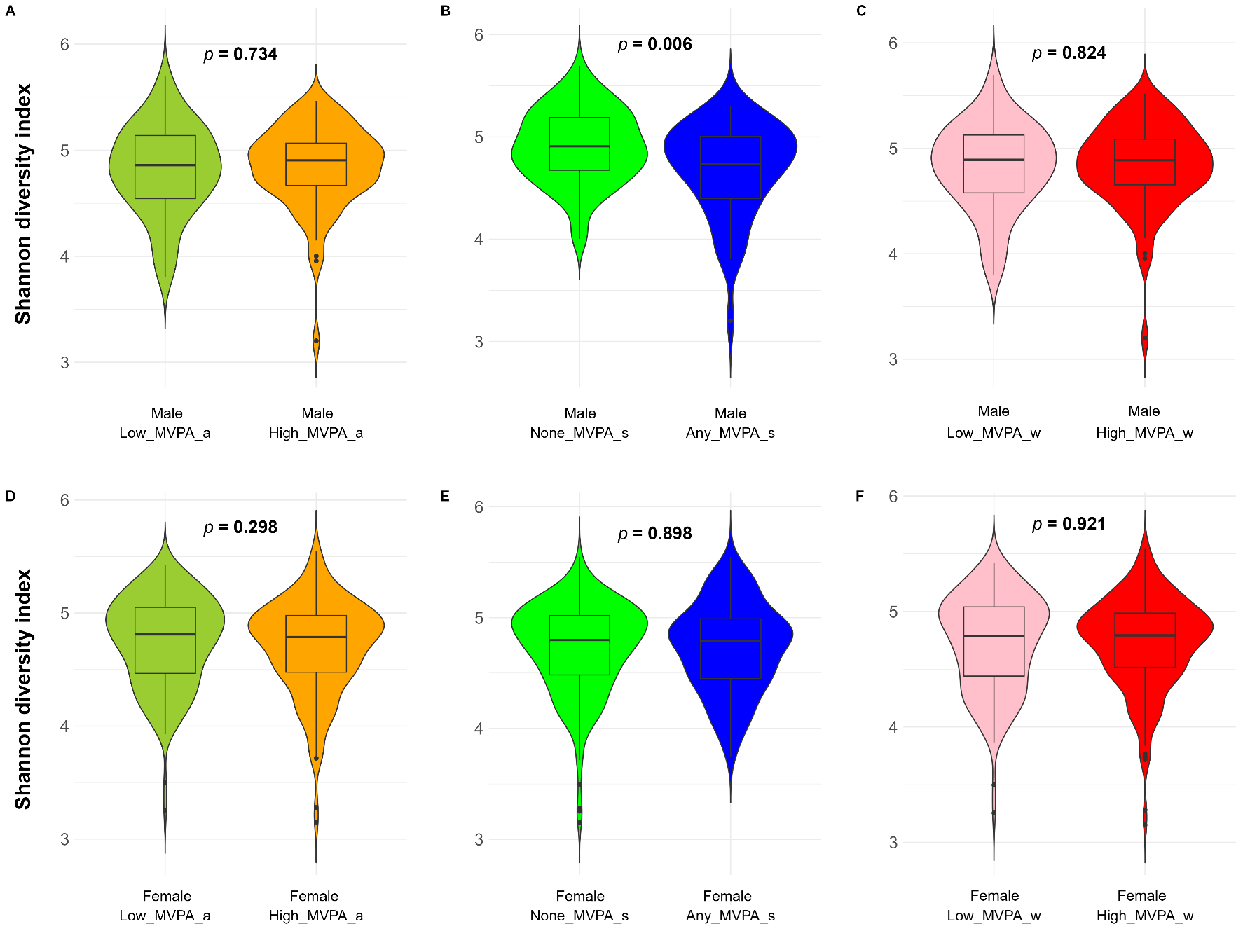


**Figure S2** Proportion of variation (R^2^) in species-level Bray–Curtis dissimilarity explained by potential confounders in permutational multivariate analysis of variance wherein total moderate to vigorous physical activity served as the main exposure. Results were derived from permutational multivariate analysis of variance on the Bray-Curtis dissimilarities among all 489 participants


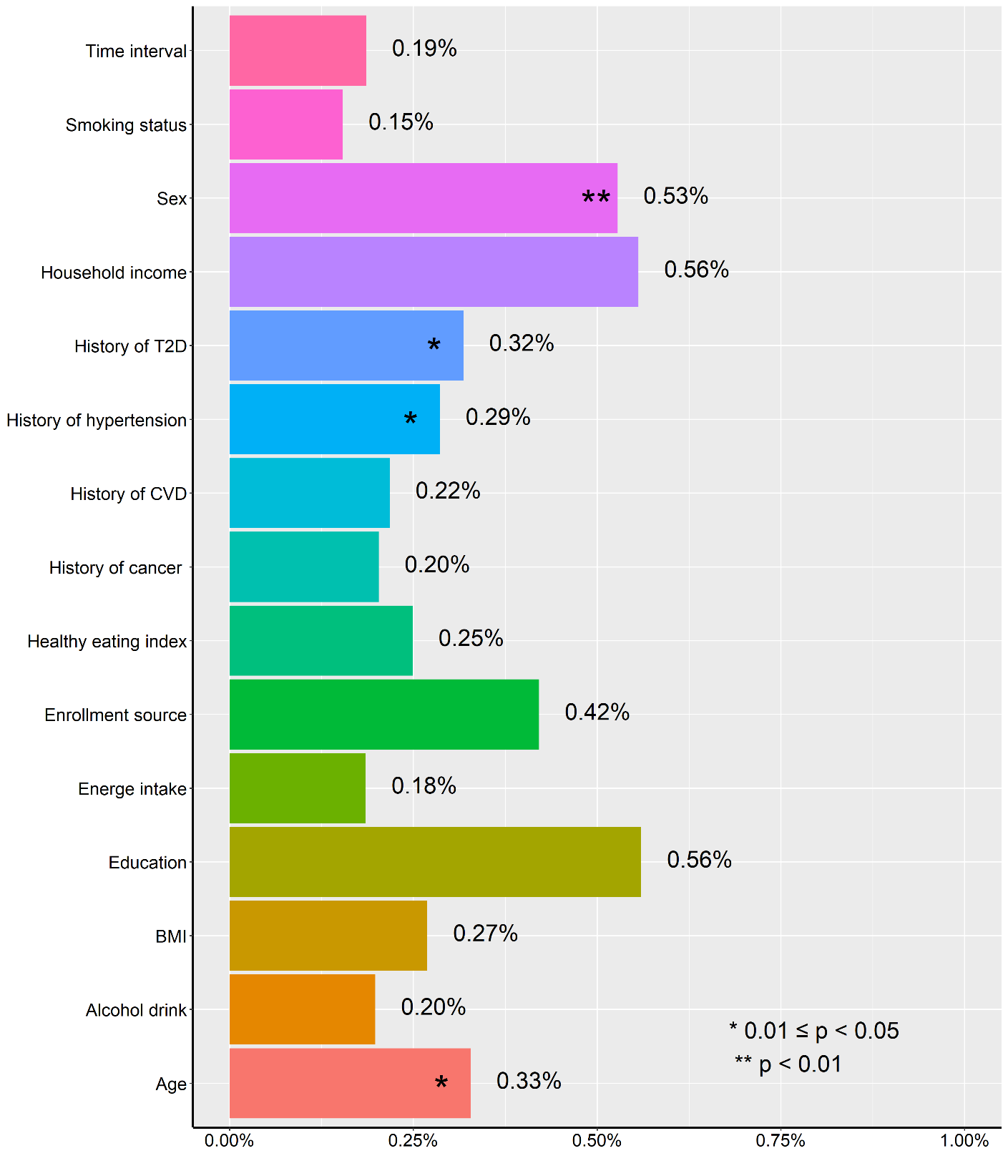


**Figure S3** Associations between physical activity measures and gut microbial species in the Southern Community Cohort Study. Centered log-ratio transformed abundance of species was regressed on PA measures, adjusted for enrollment age, sex, time interval between enrollment and stool collection, education, household income, tobacco smoking, alcohol drinking, total energy intake, healthy eating index, daily sitting hours, body mass index, and history of selected chronic diseases at baseline including cancer, cardiovascular disease, diabetes, hypertension, and **bowel movement frequency**. The significant heatmap cells are noted by FDR corrected *q-values* (* *q* <0.10) and the directions of associations are represented by colors, with red signifying positive beta coefficients and blue signifying negative beta coefficients. Phyla for species are indicated by text colors: blue for *Actinobacteriota*, red for *Bacteroidota*, dark green for *Firmicutes*, and black for *Proteobacteria*.


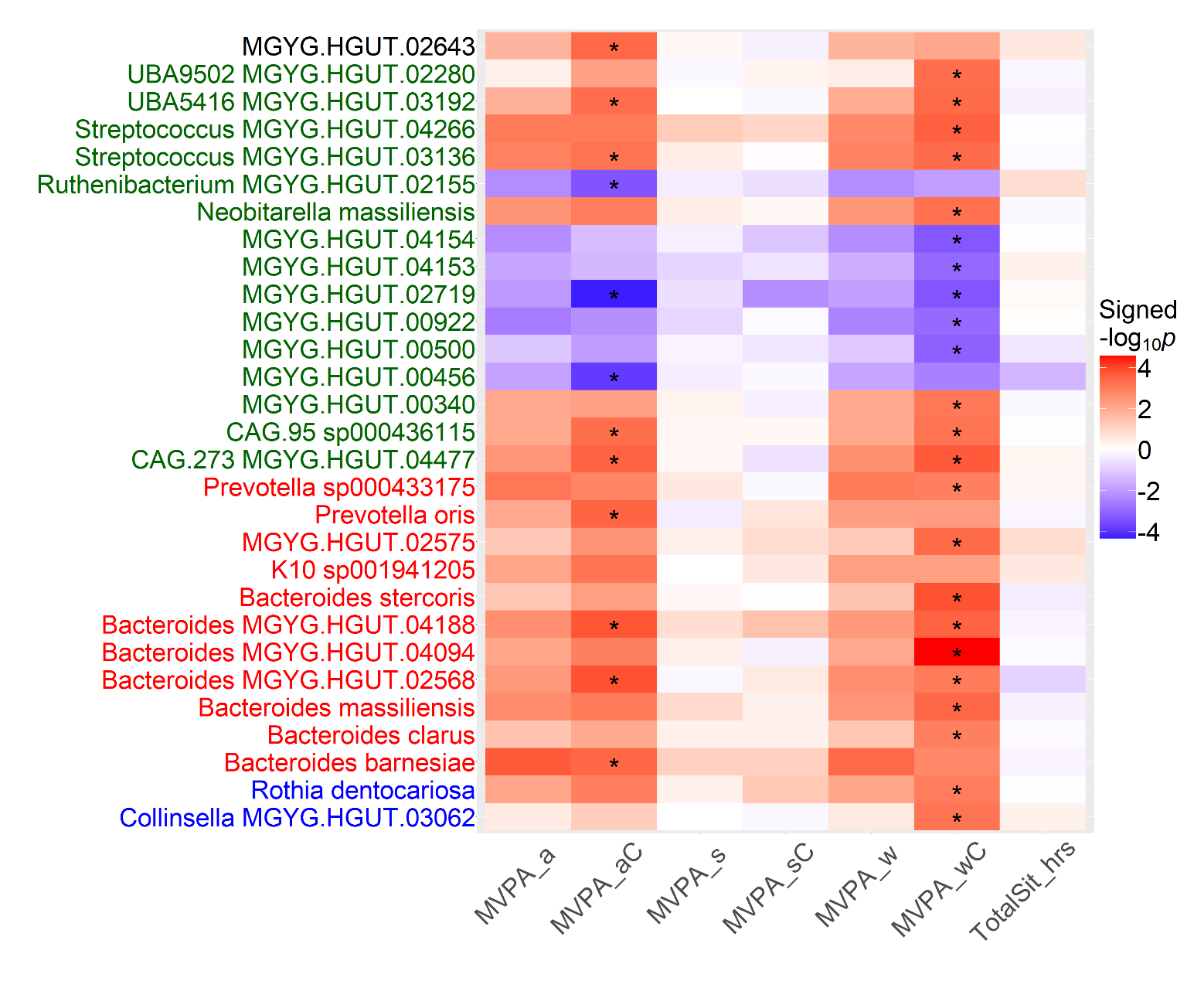


**Figure S4** Associations between physical activity measures and gut microbial species in the Southern Community Cohort Study. Centered log-ratio transformed abundance of species was regressed on PA measures, adjusted for enrollment age, sex, time interval between enrollment and stool collection, education, household income, tobacco smoking, alcohol drinking, total energy intake, healthy eating index, daily sitting hours, body mass index, and history of selected chronic diseases at baseline including cancer, cardiovascular disease, diabetes, hypertension, and **stool appearance/type**. The significant heatmap cells are noted by FDR q-values (^*^ *q* < 0.10) and the directions of associations are represented by colors, with red signifying positive beta coefficients and blue signifying negative beta coefficients. Phyla for species are indicated by text colors: blue for *Actinobacteriota*, red for *Bacteroidota*, dark green for *Firmicutes*, and black for *Proteobacteria*.


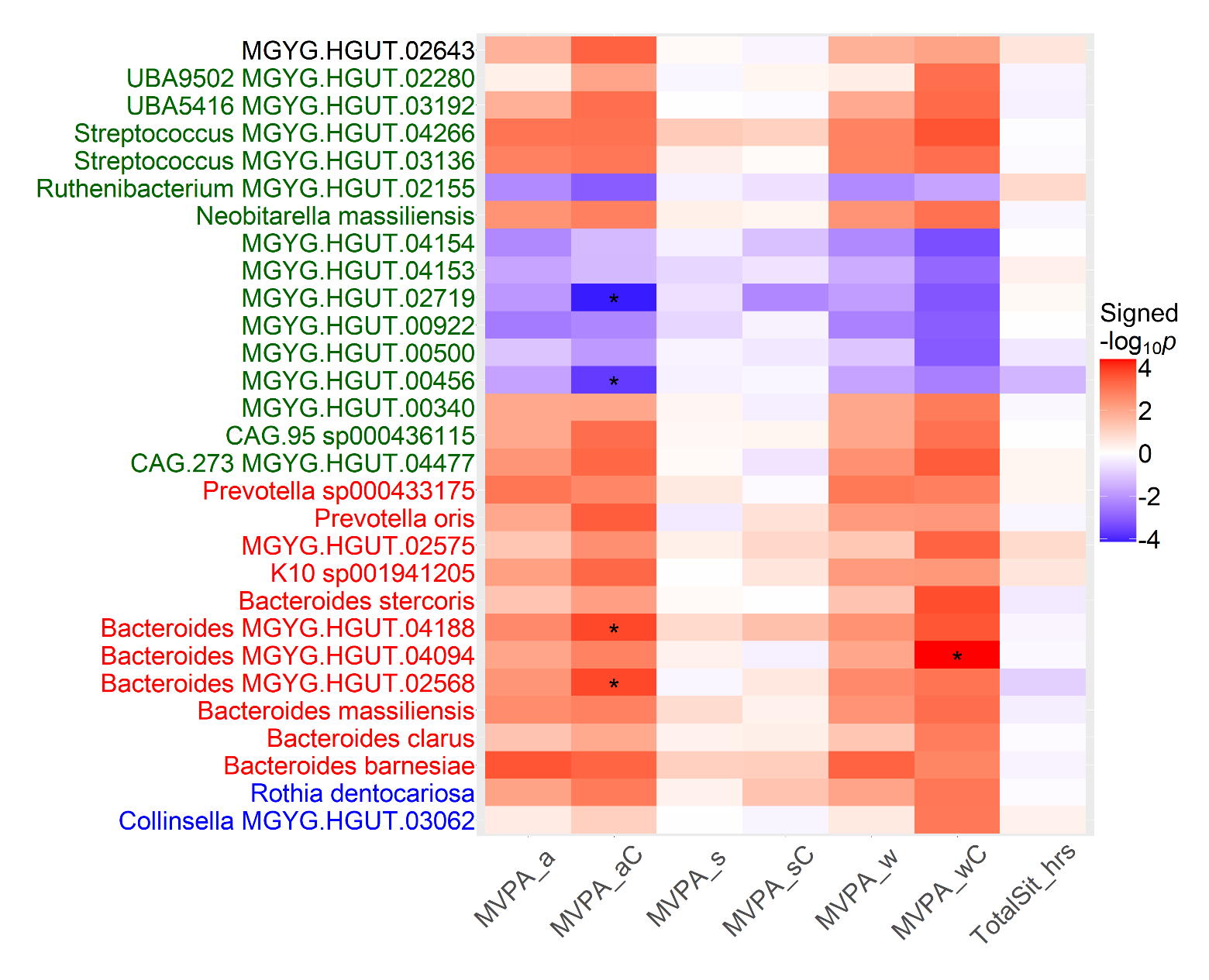


**Figure S5** Associations between physical activity measures and gut microbial metabolic pathways in the Southern Community Cohort Study. Arcsine square root transformed relative abundance of pathways was regressed on PA measures, adjusted for enrollment age, sex, time interval between enrollment and stool collection, education, household income, tobacco smoking, alcohol drinking, total energy intake, healthy eating index, daily sitting hours, body mass index, and history of selected chronic diseases at baseline including cancer, cardiovascular disease, diabetes, hypertension, and **bowel movement frequency**. The significant heatmap cells are noted by FDR corrected *q-values* (* 0.05 ≤ *q* <0.10; ** 0.01 ≤ *q* <0.05; *** *q* <0.01) and the directions of associations are represented by colors, with red signifying positive beta coefficients and blue signifying negative beta coefficients.


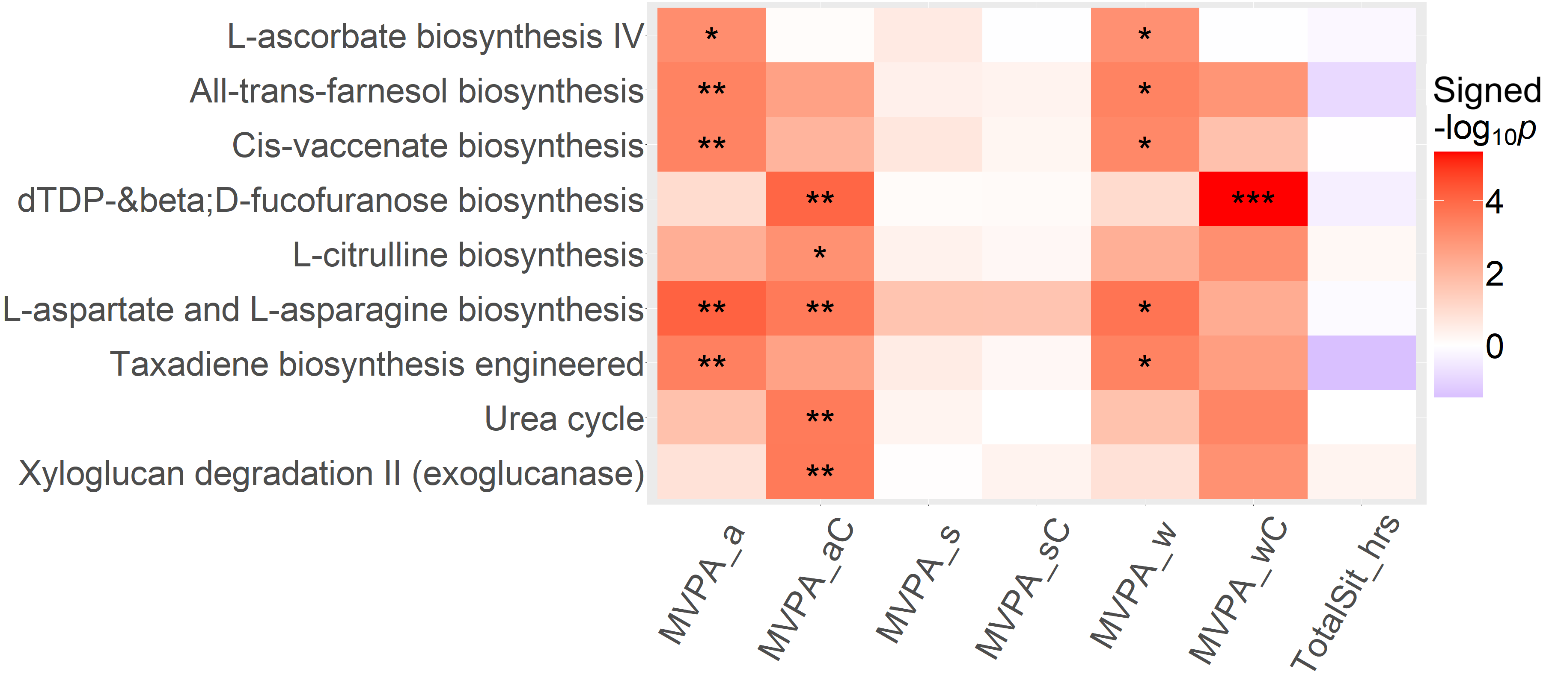


**Figure S6** Associations between physical activity measures and gut microbial metabolic pathways in the Southern Community Cohort Study. Arcsine square root transformed relative abundance of pathways was regressed on PA measures, adjusted for enrollment age, sex (only for combined analysis), time interval between enrollment and stool collection, education, household income, tobacco smoking, alcohol drinking, total energy intake, healthy eating index, daily sitting hours, body mass index, and history of selected chronic diseases at baseline including cancer, cardiovascular disease, diabetes, hypertension, and **stool appearance/type**. The significant heatmap cells are noted by FDR corrected *q*-values (* 0.05 ≤ *q* <0.10; ** 0.01 ≤ *q* <0.05; *** *q* <0.01) and the directions of associations are represented by colors, with red signifying positive beta coefficients and blue signifying negative beta coefficients.


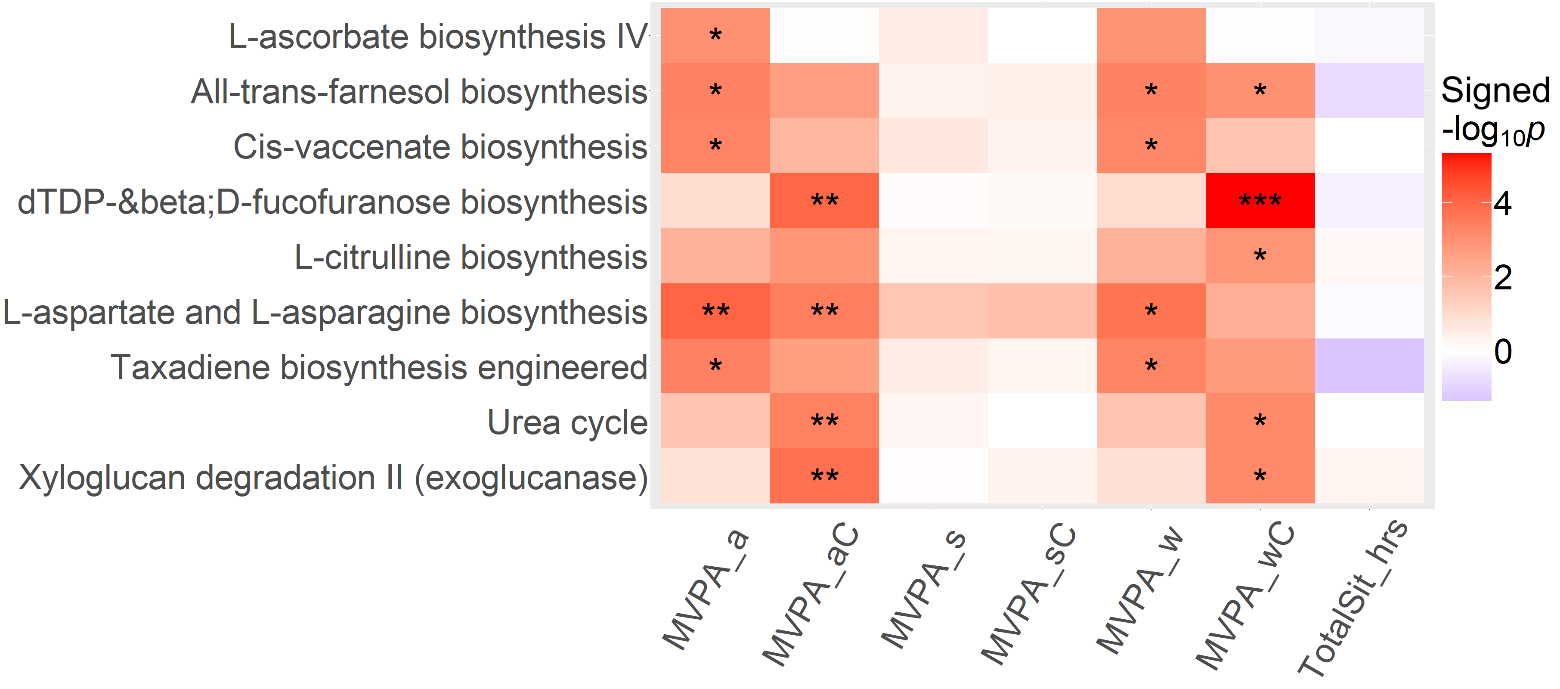


| **TABLE S1**. Abbreviations of exposures, microbial species, and metabolic pathways in this study | | |
| --- | --- | --- |
| Type | Abbreviation | Full name |
| Exposure (abbreviations defined by authors) | | |
|  | MVPA_a | Total (all) exercise/sport- and work/home-related moderate-vigorous physical activity |
|  | MVPA_aC | Categorical total (all) exercise/sport- and work/home-related moderate-vigorous physical activity by sex-specific median |
|  | MVPA_s | Exercise/sport-related moderate-vigorous physical activity |
|  | MVPA_sC | Categorical exercise/sport-related moderate-vigorous physical activity by sex-specific median |
|  | MVPA_w | Work/home-related moderate-vigorous physical activity |
|  | MVPA_wC | Categorical work/home-related moderate-vigorous physical activity by sex-specific median |
|  | TotalSit_hrs | Total hours spent in sitting activities including sitting in a car or bus, at work, watching TV or seeing movies, using a computer at home, or in other activities |
|  |  |  |
| Species (formatted as "genus species"; full names from the Unified Human Gastrointestinal Genome (UHGG) collection version 1.0 | | |
|  | Collinsella MGYG.HGUT.03062 | d__Bacteria.p__Actinobacteriota.c__Coriobacteriia.o__Coriobacteriales.f__Coriobacteriaceae.g__Collinsella.s__Collinsella MGYG.HGUT.03062 |
|  | Rothia dentocariosa | d__Bacteria.p__Actinobacteriota.c__Actinobacteria.o__Actinomycetales.f__Micrococcaceae.g__Rothia.s__Rothia_dentocariosa |
|  | Bacteroides barnesiae | d__Bacteria.p__Bacteroidota.c__Bacteroidia.o__Bacteroidales.f__Bacteroidaceae.g__Bacteroides_A.s__Bacteroides_A_barnesiae |
|  | Bacteroides clarus | d__Bacteria.p__Bacteroidota.c__Bacteroidia.o__Bacteroidales.f__Bacteroidaceae.g__Bacteroides.s__Bacteroides_clarus |
|  | Bacteroides massiliensis | d__Bacteria.p__Bacteroidota.c__Bacteroidia.o__Bacteroidales.f__Bacteroidaceae.g__Bacteroides_B.s__Bacteroides_B_massiliensis |
|  | Bacteroides MGYG.HGUT.02568 | d__Bacteria.p__Bacteroidota.c__Bacteroidia.o__Bacteroidales.f__Bacteroidaceae.g__Bacteroides_A.s__MGYG.HGUT.02568 |
|  | Bacteroides MGYG.HGUT.04094 | d__Bacteria.p__Bacteroidota.c__Bacteroidia.o__Bacteroidales.f__Bacteroidaceae.g__Bacteroides.s__MGYG.HGUT.04094 |
|  | Bacteroides MGYG.HGUT.04188 | d__Bacteria.p__Bacteroidota.c__Bacteroidia.o__Bacteroidales.f__Bacteroidaceae.g__Bacteroides.s__MGYG.HGUT.04188 |
|  | Bacteroides stercoris | d__Bacteria.p__Bacteroidota.c__Bacteroidia.o__Bacteroidales.f__Bacteroidaceae.g__Bacteroides.s__Bacteroides_stercoris |
|  | K10 sp001941205 | d__Bacteria.p__Bacteroidota.c__Bacteroidia.o__Bacteroidales.f__Muribaculaceae.g__K10.s__K10_sp001941205 |
|  | MGYG.HGUT.02575 | d__Bacteria.p__Bacteroidota.c__Bacteroidia.o__Bacteroidales.f__Bacteroidaceae.g__MGYG.HGUT.02575.s__MGYG.HGUT.02575 |
|  | Prevotella oris | d__Bacteria.p__Bacteroidota.c__Bacteroidia.o__Bacteroidales.f__Bacteroidaceae.g__Prevotella.s__Prevotella_oris |
|  | Prevotella sp000433175 | d__Bacteria.p__Bacteroidota.c__Bacteroidia.o__Bacteroidales.f__Bacteroidaceae.g__Prevotella.s__Prevotella_sp000433175 |
|  | CAG.110 sp000435995 | d__Bacteria.p__Firmicutes_A.c__Clostridia.o__Oscillospirales.f__Oscillospiraceae.g__CAG.110.s__CAG.110_sp000435995 |
|  | CAG.273 MGYG.HGUT.04477 | d__Bacteria.p__Firmicutes_A.c__Clostridia.o__TANB77.f__CAG.508.g__CAG.273.s__MGYG.HGUT.04477 |
|  | CAG.95 sp000436115 | d__Bacteria.p__Firmicutes_A.c__Clostridia.o__Lachnospirales.f__Lachnospiraceae.g__CAG.95.s__CAG.95_sp000436115 |
|  | MGYG.HGUT.00340 | d__Bacteria.p__Firmicutes_A.c__Clostridia.o__Oscillospirales.f__Acutalibacteraceae.g__Clostridium_E.s__MGYG.HGUT.00340 |
|  | MGYG.HGUT.00456 | d__Bacteria.p__Firmicutes_A.c__Clostridia.o__Oscillospirales.f__Acutalibacteraceae.g__MGYG.HGUT.00456.s__MGYG.HGUT.00456 |
|  | MGYG.HGUT.00500 | d__Bacteria.p__Firmicutes_A.c__Clostridia.o__Oscillospirales.f__Oscillospiraceae.g__CAG.110.s__MGYG.HGUT.00500 |
|  | MGYG.HGUT.00922 | d__Bacteria.p__Firmicutes_A.c__Clostridia.o__Christensenellales.f__MGYG.HGUT.00922.g__MGYG.HGUT.00922.s__MGYG.HGUT.00922 |
|  | MGYG.HGUT.02709 | d__Bacteria.p__Firmicutes_A.c__Clostridia.o__Oscillospirales.f__Acutalibacteraceae.g__MGYG.HGUT.02709.s__MGYG.HGUT.02709 |
|  | MGYG.HGUT.02719 | d__Bacteria.p__Firmicutes_A.c__Clostridia.o__MGYG.HGUT.02719.f__MGYG.HGUT.02719.g__MGYG.HGUT.02719.s__MGYG.HGUT.02719 |
|  | MGYG.HGUT.04153 | d__Bacteria.p__Firmicutes_A.c__Clostridia.o__Christensenellales.f__UBA1750.g__UBA7102.s__MGYG.HGUT.04153 |
|  | MGYG.HGUT.04154 | d__Bacteria.p__Firmicutes_A.c__Clostridia.o__Christensenellales.f__CAG.74.g__MGYG.HGUT.04154.s__MGYG.HGUT.04154 |
|  | Neobitarella massiliensis | d__Bacteria.p__Firmicutes_A.c__Clostridia.o__Oscillospirales.f__Ruminococcaceae.g__Neobitarella.s__Neobitarella_massiliensis |
|  | Ruthenibacterium MGYG.HGUT.02155 | d__Bacteria.p__Firmicutes_A.c__Clostridia.o__Oscillospirales.f__Ruminococcaceae.g__Ruthenibacterium.s__MGYG.HGUT.02155 |
|  | Streptococcus MGYG.HGUT.03136 | d__Bacteria.p__Firmicutes.c__Bacilli.o__Lactobacillales.f__Streptococcaceae.g__Streptococcus.s__MGYG.HGUT.03136 |
|  | Streptococcus MGYG.HGUT.04266 | d__Bacteria.p__Firmicutes.c__Bacilli.o__Lactobacillales.f__Streptococcaceae.g__Streptococcus.s__MGYG.HGUT.04266 |
|  | UBA5416 MGYG.HGUT.03192 | d__Bacteria.p__Firmicutes_A.c__Clostridia.o__Lachnospirales.f__Lachnospiraceae.g__UBA5416.s__MGYG.HGUT.03192 |
|  | UBA9502 MGYG.HGUT.02280 | d__Bacteria.p__Firmicutes_A.c__Clostridia.o__Lachnospirales.f__Lachnospiraceae.g__UBA9502.s__MGYG.HGUT.02280 |
|  | MGYG.HGUT.03211 | d__Bacteria.p__Firmicutes_A.c__Clostridia.o__Oscillospirales.f__Acutalibacteraceae.g__MGYG.HGUT.03211.s__MGYG.HGUT.03211 |
|  | MGYG.HGUT.02643 | d__Bacteria.p__Proteobacteria.c__Gammaproteobacteria.o__Burkholderiales.f__Burkholderiaceae.g__Turicimonas.s__MGYG.HGUT.02643 |
|  |  |  |
| Pathway (full names from the MetaCyc database (version 24.0)) | | |
|  | All-trans-farnesol biosynthesis | PWY.6859..all.trans.farnesol.biosynthesis |
|  | Cis-vaccenate biosynthesis | PWY.5973..cis.vaccenate.biosynthesis |
|  | dTDP-&beta;D-fucofuranose biosynthesis | PWY.7312..dTDP..beta..D.fucofuranose.biosynthesis |
|  | L-ascorbate biosynthesis IV | PWY3DJ.35471..L.ascorbate.biosynthesis.IV..animals..D.glucuronate.pathway. |
|  | L-aspartate and L-asparagine biosynthesis | ASPASN.PWY..superpathway.of.L.aspartate.and.L.asparagine.biosynthesis |
|  | L-citrulline biosynthesis | CITRULBIO.PWY..L.citrulline.biosynthesis |
|  | Taxadiene biosynthesis engineered | PWY.7392..taxadiene.biosynthesis..engineered. |
|  | Urea cycle | PWY.4984..urea.cycle |
|  | Xyloglucan degradation II (exoglucanase) | PWY.6807..xyloglucan.degradation.II..exoglucanase. |
| CAG, Co-abundance Groups; MGYG-HGUT, Metagenomic Gut Yielding Genome-Human Gut Unified Taxonomy; PWY, pathway; UBA, Uncultivated Bacteria and Archaea; microbial taxonomy: d__, domain; p__, phylum; c__, class; o__, order; f__, family; g__, genus; s__, species. | | |

| **Supplemental Table 2**. Microbial species associated with physical activity measures in all selected SCCS participants | | | | | | | | |
| --- | --- | --- | --- | --- | --- | --- | --- | --- |
| Species | Phylum | Prevalence | Median CLR-transformed abundance | Beta^a^ | Se^a^ | *P*^a^ | fdr^a^ | PA measure |
| Collinsella MGYG.HGUT.03062 | Actinobacteriota | 74.2 | 2.533 | 0.0016 | 0.0017 | 0.3316 | 0.713 | MVPA_a |
| Rothia dentocariosa | Actinobacteriota | 26.6 | -4.625 | 0.0037 | 0.0014 | 0.0067 | 0.253 | MVPA_a |
| Bacteroides barnesiae | Bacteroidota | 93.9 | 3.183 | 0.0041 | 0.0011 | 2.04E-04 | 0.192 | MVPA_a |
| Bacteroides clarus | Bacteroidota | 95.9 | 3.023 | 0.0021 | 0.001 | 0.0375 | 0.419 | MVPA_a |
| Bacteroides massiliensis | Bacteroidota | 99.0 | 5.721 | 0.003 | 0.001 | 0.0021 | 0.204 | MVPA_a |
| Bacteroides MGYG.HGUT.02568 | Bacteroidota | 44.0 | -3.894 | 0.0044 | 0.0015 | 0.004 | 0.220 | MVPA_a |
| Bacteroides MGYG.HGUT.04094 | Bacteroidota | 83.0 | 1.527 | 0.0035 | 0.0013 | 0.0075 | 0.269 | MVPA_a |
| Bacteroides MGYG.HGUT.04188 | Bacteroidota | 90.2 | 1.852 | 0.0035 | 0.0011 | 0.0021 | 0.204 | MVPA_a |
| Bacteroides stercoris | Bacteroidota | 99.4 | 5.544 | 0.0024 | 0.0012 | 0.0395 | 0.421 | MVPA_a |
| K10 sp001941205 | Bacteroidota | 22.9 | -4.651 | 0.0035 | 0.0013 | 0.0061 | 0.245 | MVPA_a |
| MGYG.HGUT.02575 | Bacteroidota | 86.1 | 1.937 | 0.0028 | 0.0014 | 0.0412 | 0.422 | MVPA_a |
| Prevotella oris | Bacteroidota | 95.3 | 2.037 | 0.002 | 0.0008 | 0.0108 | 0.295 | MVPA_a |
| Prevotella sp000433175 | Bacteroidota | 58.5 | 0.239 | 0.0051 | 0.0015 | 7.27E-04 | 0.204 | MVPA_a |
| CAG.110 sp000435995 | Firmicutes | 40.7 | -3.903 | -0.0044 | 0.0018 | 0.0181 | 0.321 | MVPA_a |
| CAG.273 MGYG.HGUT.04477 | Firmicutes | 59.3 | 0.305 | 0.0045 | 0.0015 | 0.0031 | 0.209 | MVPA_a |
| CAG.95 sp000436115 | Firmicutes | 93.3 | 1.664 | 0.0021 | 0.0008 | 0.0106 | 0.295 | MVPA_a |
| MGYG.HGUT.00340 | Firmicutes | 82.2 | 1.752 | 0.0035 | 0.0013 | 0.0087 | 0.285 | MVPA_a |
| MGYG.HGUT.00456 | Firmicutes | 76.7 | 1.015 | -0.0029 | 0.0012 | 0.0142 | 0.306 | MVPA_a |
| MGYG.HGUT.00500 | Firmicutes | 66.3 | 0.391 | -0.0023 | 0.0013 | 0.0707 | 0.443 | MVPA_a |
| MGYG.HGUT.00922 | Firmicutes | 48.9 | -3.070 | -0.0047 | 0.0015 | 0.0026 | 0.207 | MVPA_a |
| MGYG.HGUT.02709 | Firmicutes | 60.7 | 0.316 | -0.0028 | 0.0014 | 0.0433 | 0.428 | MVPA_a |
| MGYG.HGUT.02719 | Firmicutes | 95.5 | 1.953 | -0.0017 | 0.0007 | 0.01 | 0.295 | MVPA_a |
| MGYG.HGUT.03211 | Firmicutes | 71.6 | 1.772 | -0.004 | 0.0015 | 0.0088 | 0.285 | MVPA_a |
| MGYG.HGUT.04153 | Firmicutes | 45.8 | -3.686 | -0.004 | 0.0017 | 0.0193 | 0.328 | MVPA_a |
| MGYG.HGUT.04154 | Firmicutes | 28.6 | -4.406 | -0.0037 | 0.0013 | 0.006 | 0.245 | MVPA_a |
| Neobitarella massiliensis | Firmicutes | 99.4 | 4.516 | 0.0021 | 0.0007 | 0.003 | 0.209 | MVPA_a |
| Ruthenibacterium MGYG.HGUT.02155 | Firmicutes | 61.3 | 0.528 | -0.005 | 0.0018 | 0.0049 | 0.245 | MVPA_a |
| Streptococcus MGYG.HGUT.03136 | Firmicutes | 83.6 | 1.791 | 0.0043 | 0.0013 | 0.0011 | 0.204 | MVPA_a |
| Streptococcus MGYG.HGUT.04266 | Firmicutes | 71.6 | 1.052 | 0.005 | 0.0015 | 8.82E-04 | 0.204 | MVPA_a |
| UBA5416 MGYG.HGUT.03192 | Firmicutes | 99.8 | 2.823 | 0.0012 | 0.0005 | 0.0135 | 0.303 | MVPA_a |
| UBA9502 MGYG.HGUT.02280 | Firmicutes | 80.2 | 1.120 | 0.0009 | 0.0012 | 0.4574 | 0.773 | MVPA_a |
| MGYG.HGUT.02643 | Proteobacteria | 27.6 | -4.494 | 0.0032 | 0.0014 | 0.0214 | 0.329 | MVPA_a |
| Collinsella MGYG.HGUT.03062 | Actinobacteriota | 74.2 | 2.533 | 0.605 | 0.3284 | 0.0661 | 0.405 | MVPA_aC |
| Rothia dentocariosa | Actinobacteriota | 26.6 | -4.625 | 0.8806 | 0.2644 | 9.35E-04 | 0.098 | MVPA_aC |
| Bacteroides barnesiae | Bacteroidota | 93.9 | 3.183 | 0.7686 | 0.2128 | 3.36E-04 | 0.082 | MVPA_aC |
| Bacteroides clarus | Bacteroidota | 95.9 | 3.023 | 0.5233 | 0.1987 | 0.0087 | 0.235 | MVPA_aC |
| Bacteroides massiliensis | Bacteroidota | 99.0 | 5.721 | 0.6202 | 0.1857 | 9.07E-04 | 0.098 | MVPA_aC |
| Bacteroides MGYG.HGUT.02568 | Bacteroidota | 44.0 | -3.894 | 1.1145 | 0.292 | 1.54E-04 | 0.076 | MVPA_aC |
| Bacteroides MGYG.HGUT.04094 | Bacteroidota | 83.0 | 1.527 | 0.8199 | 0.2495 | 0.0011 | 0.098 | MVPA_aC |
| Bacteroides MGYG.HGUT.04188 | Bacteroidota | 90.2 | 1.852 | 0.8234 | 0.2164 | 1.61E-04 | 0.076 | MVPA_aC |
| Bacteroides stercoris | Bacteroidota | 99.4 | 5.544 | 0.6475 | 0.2286 | 0.0048 | 0.207 | MVPA_aC |
| K10 sp001941205 | Bacteroidota | 22.9 | -4.651 | 0.8584 | 0.2467 | 5.48E-04 | 0.082 | MVPA_aC |
| MGYG.HGUT.02575 | Bacteroidota | 86.1 | 1.937 | 0.8016 | 0.2647 | 0.0026 | 0.158 | MVPA_aC |
| Prevotella oris | Bacteroidota | 95.3 | 2.037 | 0.5428 | 0.1508 | 3.53E-04 | 0.082 | MVPA_aC |
| Prevotella sp000433175 | Bacteroidota | 58.5 | 0.239 | 0.938 | 0.2905 | 0.0013 | 0.114 | MVPA_aC |
| CAG.110 sp000435995 | Firmicutes | 40.7 | -3.903 | -1.173 | 0.3569 | 0.0011 | 0.098 | MVPA_aC |
| CAG.273 MGYG.HGUT.04477 | Firmicutes | 59.3 | 0.305 | 1.0629 | 0.2913 | 2.93E-04 | 0.082 | MVPA_aC |
| CAG.95 sp000436115 | Firmicutes | 93.3 | 1.664 | 0.5495 | 0.1583 | 5.65E-04 | 0.082 | MVPA_aC |
| MGYG.HGUT.00340 | Firmicutes | 82.2 | 1.752 | 0.7146 | 0.2603 | 0.0063 | 0.216 | MVPA_aC |
| MGYG.HGUT.00456 | Firmicutes | 76.7 | 1.015 | -0.8757 | 0.2266 | 1.27E-04 | 0.076 | MVPA_aC |
| MGYG.HGUT.00500 | Firmicutes | 66.3 | 0.391 | -0.6311 | 0.2437 | 0.0099 | 0.239 | MVPA_aC |
| MGYG.HGUT.00922 | Firmicutes | 48.9 | -3.070 | -0.8251 | 0.2999 | 0.0062 | 0.216 | MVPA_aC |
| MGYG.HGUT.02709 | Firmicutes | 60.7 | 0.316 | -0.8965 | 0.2715 | 0.001 | 0.098 | MVPA_aC |
| MGYG.HGUT.02719 | Firmicutes | 95.5 | 1.953 | -0.5251 | 0.1283 | 5.04E-05 | 0.076 | MVPA_aC |
| MGYG.HGUT.03211 | Firmicutes | 71.6 | 1.772 | -1.0094 | 0.297 | 7.36E-04 | 0.098 | MVPA_aC |
| MGYG.HGUT.04153 | Firmicutes | 45.8 | -3.686 | -0.6838 | 0.3327 | 0.0404 | 0.362 | MVPA_aC |
| MGYG.HGUT.04154 | Firmicutes | 28.6 | -4.406 | -0.5087 | 0.2603 | 0.0513 | 0.369 | MVPA_aC |
| Neobitarella massiliensis | Firmicutes | 99.4 | 4.516 | 0.4574 | 0.138 | 9.86E-04 | 0.098 | MVPA_aC |
| Ruthenibacterium MGYG.HGUT.02155 | Firmicutes | 61.3 | 0.528 | -1.2328 | 0.3444 | 3.80E-04 | 0.082 | MVPA_aC |
| Streptococcus MGYG.HGUT.03136 | Firmicutes | 83.6 | 1.791 | 0.879 | 0.2519 | 5.29E-04 | 0.082 | MVPA_aC |
| Streptococcus MGYG.HGUT.04266 | Firmicutes | 71.6 | 1.052 | 0.9699 | 0.2881 | 8.23E-04 | 0.098 | MVPA_aC |
| UBA5416 MGYG.HGUT.03192 | Firmicutes | 99.8 | 2.823 | 0.3324 | 0.0942 | 4.57E-04 | 0.082 | MVPA_aC |
| UBA9502 MGYG.HGUT.02280 | Firmicutes | 80.2 | 1.120 | 0.6348 | 0.235 | 0.0072 | 0.219 | MVPA_aC |
| MGYG.HGUT.02643 | Proteobacteria | 27.6 | -4.494 | 0.934 | 0.2671 | 5.16E-04 | 0.082 | MVPA_aC |
| Collinsella MGYG.HGUT.03062 | Actinobacteriota | 74.2 | 2.533 | 0.0001 | 0.0107 | 0.9935 | 0.998 | MVPA_s |
| Rothia dentocariosa | Actinobacteriota | 26.6 | -4.625 | 0.0064 | 0.0087 | 0.4584 | 0.982 | MVPA_s |
| Bacteroides barnesiae | Bacteroidota | 93.9 | 3.183 | 0.0126 | 0.007 | 0.0716 | 0.980 | MVPA_s |
| Bacteroides clarus | Bacteroidota | 95.9 | 3.023 | 0.0051 | 0.0065 | 0.4329 | 0.982 | MVPA_s |
| Bacteroides massiliensis | Bacteroidota | 99.0 | 5.721 | 0.0093 | 0.0061 | 0.1253 | 0.980 | MVPA_s |
| Bacteroides MGYG.HGUT.02568 | Bacteroidota | 44.0 | -3.894 | -0.0036 | 0.0096 | 0.7054 | 0.994 | MVPA_s |
| Bacteroides MGYG.HGUT.04094 | Bacteroidota | 83.0 | 1.527 | 0.0067 | 0.0082 | 0.4162 | 0.982 | MVPA_s |
| Bacteroides MGYG.HGUT.04188 | Bacteroidota | 90.2 | 1.852 | 0.0105 | 0.0071 | 0.1408 | 0.980 | MVPA_s |
| Bacteroides stercoris | Bacteroidota | 99.4 | 5.544 | 0.0031 | 0.0075 | 0.678 | 0.994 | MVPA_s |
| K10 sp001941205 | Bacteroidota | 22.9 | -4.651 | 0.0005 | 0.0081 | 0.9467 | 0.997 | MVPA_s |
| MGYG.HGUT.02575 | Bacteroidota | 86.1 | 1.937 | 0.0073 | 0.0087 | 0.4003 | 0.982 | MVPA_s |
| Prevotella oris | Bacteroidota | 95.3 | 2.037 | -0.004 | 0.005 | 0.4237 | 0.982 | MVPA_s |
| Prevotella sp000433175 | Bacteroidota | 58.5 | 0.239 | 0.0102 | 0.0095 | 0.2861 | 0.980 | MVPA_s |
| CAG.110 sp000435995 | Firmicutes | 40.7 | -3.903 | -0.0024 | 0.0117 | 0.836 | 0.997 | MVPA_s |
| CAG.273 MGYG.HGUT.04477 | Firmicutes | 59.3 | 0.305 | 0.0042 | 0.0096 | 0.661 | 0.994 | MVPA_s |
| CAG.95 sp000436115 | Firmicutes | 93.3 | 1.664 | 0.0024 | 0.0052 | 0.6388 | 0.992 | MVPA_s |
| MGYG.HGUT.00340 | Firmicutes | 82.2 | 1.752 | 0.0054 | 0.0085 | 0.525 | 0.984 | MVPA_s |
| MGYG.HGUT.00456 | Firmicutes | 76.7 | 1.015 | -0.0055 | 0.0075 | 0.4617 | 0.982 | MVPA_s |
| MGYG.HGUT.00500 | Firmicutes | 66.3 | 0.391 | -0.0046 | 0.008 | 0.5638 | 0.984 | MVPA_s |
| MGYG.HGUT.00922 | Firmicutes | 48.9 | -3.070 | -0.0137 | 0.0098 | 0.1628 | 0.980 | MVPA_s |
| MGYG.HGUT.02709 | Firmicutes | 60.7 | 0.316 | -0.0082 | 0.0089 | 0.3583 | 0.980 | MVPA_s |
| MGYG.HGUT.02719 | Firmicutes | 95.5 | 1.953 | -0.0049 | 0.0042 | 0.2507 | 0.980 | MVPA_s |
| MGYG.HGUT.03211 | Firmicutes | 71.6 | 1.772 | -0.0109 | 0.0097 | 0.2633 | 0.980 | MVPA_s |
| MGYG.HGUT.04153 | Firmicutes | 45.8 | -3.686 | -0.0152 | 0.0108 | 0.1608 | 0.980 | MVPA_s |
| MGYG.HGUT.04154 | Firmicutes | 28.6 | -4.406 | -0.0056 | 0.0085 | 0.5119 | 0.984 | MVPA_s |
| Neobitarella massiliensis | Firmicutes | 99.4 | 4.516 | 0.0039 | 0.0045 | 0.3879 | 0.982 | MVPA_s |
| Ruthenibacterium MGYG.HGUT.02155 | Firmicutes | 61.3 | 0.528 | -0.0087 | 0.0113 | 0.4405 | 0.982 | MVPA_s |
| Streptococcus MGYG.HGUT.03136 | Firmicutes | 83.6 | 1.791 | 0.0076 | 0.0083 | 0.3557 | 0.980 | MVPA_s |
| Streptococcus MGYG.HGUT.04266 | Firmicutes | 71.6 | 1.052 | 0.0177 | 0.0094 | 0.0609 | 0.980 | MVPA_s |
| UBA5416 MGYG.HGUT.03192 | Firmicutes | 99.8 | 2.823 | 0.0001 | 0.0031 | 0.9743 | 0.998 | MVPA_s |
| UBA9502 MGYG.HGUT.02280 | Firmicutes | 80.2 | 1.120 | -0.003 | 0.0077 | 0.6945 | 0.994 | MVPA_s |
| MGYG.HGUT.02643 | Proteobacteria | 27.6 | -4.494 | 0.0032 | 0.0088 | 0.7188 | 0.994 | MVPA_s |
| Collinsella MGYG.HGUT.03062 | Actinobacteriota | 74.2 | 2.533 | -0.1616 | 0.3672 | 0.6601 | 0.928 | MVPA_sC |
| Rothia dentocariosa | Actinobacteriota | 26.6 | -4.625 | 0.5938 | 0.2968 | 0.046 | 0.621 | MVPA_sC |
| Bacteroides barnesiae | Bacteroidota | 93.9 | 3.183 | 0.4355 | 0.2395 | 0.0697 | 0.626 | MVPA_sC |
| Bacteroides clarus | Bacteroidota | 95.9 | 3.023 | 0.1816 | 0.2229 | 0.4158 | 0.835 | MVPA_sC |
| Bacteroides massiliensis | Bacteroidota | 99.0 | 5.721 | 0.1542 | 0.2093 | 0.4615 | 0.853 | MVPA_sC |
| Bacteroides MGYG.HGUT.02568 | Bacteroidota | 44.0 | -3.894 | 0.3436 | 0.3301 | 0.2984 | 0.791 | MVPA_sC |
| Bacteroides MGYG.HGUT.04094 | Bacteroidota | 83.0 | 1.527 | -0.176 | 0.2811 | 0.5316 | 0.873 | MVPA_sC |
| Bacteroides MGYG.HGUT.04188 | Bacteroidota | 90.2 | 1.852 | 0.5332 | 0.2437 | 0.0291 | 0.561 | MVPA_sC |
| Bacteroides stercoris | Bacteroidota | 99.4 | 5.544 | -0.0085 | 0.2569 | 0.9738 | 0.996 | MVPA_sC |
| K10 sp001941205 | Bacteroidota | 22.9 | -4.651 | 0.3076 | 0.278 | 0.2691 | 0.787 | MVPA_sC |
| MGYG.HGUT.02575 | Bacteroidota | 86.1 | 1.937 | 0.457 | 0.2971 | 0.1247 | 0.718 | MVPA_sC |
| Prevotella oris | Bacteroidota | 95.3 | 2.037 | 0.2044 | 0.1701 | 0.2301 | 0.781 | MVPA_sC |
| Prevotella sp000433175 | Bacteroidota | 58.5 | 0.239 | -0.0912 | 0.3273 | 0.7805 | 0.961 | MVPA_sC |
| CAG.110 sp000435995 | Firmicutes | 40.7 | -3.903 | -0.2136 | 0.4022 | 0.5957 | 0.887 | MVPA_sC |
| CAG.273 MGYG.HGUT.04477 | Firmicutes | 59.3 | 0.305 | -0.3386 | 0.3288 | 0.3036 | 0.791 | MVPA_sC |
| CAG.95 sp000436115 | Firmicutes | 93.3 | 1.664 | 0.0784 | 0.1786 | 0.6609 | 0.928 | MVPA_sC |
| MGYG.HGUT.00340 | Firmicutes | 82.2 | 1.752 | -0.1839 | 0.2923 | 0.5295 | 0.873 | MVPA_sC |
| MGYG.HGUT.00456 | Firmicutes | 76.7 | 1.015 | -0.1035 | 0.2565 | 0.6868 | 0.932 | MVPA_sC |
| MGYG.HGUT.00500 | Firmicutes | 66.3 | 0.391 | -0.2832 | 0.2732 | 0.3004 | 0.791 | MVPA_sC |
| MGYG.HGUT.00922 | Firmicutes | 48.9 | -3.070 | -0.1306 | 0.3369 | 0.6984 | 0.935 | MVPA_sC |
| MGYG.HGUT.02709 | Firmicutes | 60.7 | 0.316 | -0.5784 | 0.3049 | 0.0584 | 0.626 | MVPA_sC |
| MGYG.HGUT.02719 | Firmicutes | 95.5 | 1.953 | -0.4039 | 0.1443 | 0.0054 | 0.465 | MVPA_sC |
| MGYG.HGUT.03211 | Firmicutes | 71.6 | 1.772 | -0.2439 | 0.3349 | 0.4667 | 0.855 | MVPA_sC |
| MGYG.HGUT.04153 | Firmicutes | 45.8 | -3.686 | -0.4106 | 0.372 | 0.2703 | 0.787 | MVPA_sC |
| MGYG.HGUT.04154 | Firmicutes | 28.6 | -4.406 | -0.5206 | 0.2903 | 0.0735 | 0.626 | MVPA_sC |
| Neobitarella massiliensis | Firmicutes | 99.4 | 4.516 | 0.0807 | 0.1555 | 0.604 | 0.891 | MVPA_sC |
| Ruthenibacterium MGYG.HGUT.02155 | Firmicutes | 61.3 | 0.528 | -0.4739 | 0.3884 | 0.223 | 0.781 | MVPA_sC |
| Streptococcus MGYG.HGUT.03136 | Firmicutes | 83.6 | 1.791 | 0.0615 | 0.2843 | 0.8289 | 0.977 | MVPA_sC |
| Streptococcus MGYG.HGUT.04266 | Firmicutes | 71.6 | 1.052 | 0.5344 | 0.3239 | 0.0997 | 0.679 | MVPA_sC |
| UBA5416 MGYG.HGUT.03192 | Firmicutes | 99.8 | 2.823 | -0.0321 | 0.1063 | 0.7628 | 0.955 | MVPA_sC |
| UBA9502 MGYG.HGUT.02280 | Firmicutes | 80.2 | 1.120 | 0.1652 | 0.2638 | 0.5314 | 0.873 | MVPA_sC |
| MGYG.HGUT.02643 | Proteobacteria | 27.6 | -4.494 | -0.1768 | 0.3014 | 0.5578 | 0.884 | MVPA_sC |
| Collinsella MGYG.HGUT.03062 | Actinobacteriota | 74.2 | 2.533 | 0.0018 | 0.0018 | 0.3134 | 0.708 | MVPA_w |
| Rothia dentocariosa | Actinobacteriota | 26.6 | -4.625 | 0.0038 | 0.0014 | 0.007 | 0.246 | MVPA_w |
| Bacteroides barnesiae | Bacteroidota | 93.9 | 3.183 | 0.0041 | 0.0011 | 3.69E-04 | 0.188 | MVPA_w |
| Bacteroides clarus | Bacteroidota | 95.9 | 3.023 | 0.0022 | 0.0011 | 0.0421 | 0.444 | MVPA_w |
| Bacteroides massiliensis | Bacteroidota | 99.0 | 5.721 | 0.0029 | 0.001 | 0.0032 | 0.212 | MVPA_w |
| Bacteroides MGYG.HGUT.02568 | Bacteroidota | 44.0 | -3.894 | 0.0048 | 0.0016 | 0.0023 | 0.212 | MVPA_w |
| Bacteroides MGYG.HGUT.04094 | Bacteroidota | 83.0 | 1.527 | 0.0036 | 0.0013 | 0.0082 | 0.267 | MVPA_w |
| Bacteroides MGYG.HGUT.04188 | Bacteroidota | 90.2 | 1.852 | 0.0034 | 0.0012 | 0.0032 | 0.212 | MVPA_w |
| Bacteroides stercoris | Bacteroidota | 99.4 | 5.544 | 0.0025 | 0.0012 | 0.0382 | 0.436 | MVPA_w |
| K10 sp001941205 | Bacteroidota | 22.9 | -4.651 | 0.0038 | 0.0013 | 0.0045 | 0.246 | MVPA_w |
| MGYG.HGUT.02575 | Bacteroidota | 86.1 | 1.937 | 0.0028 | 0.0014 | 0.0473 | 0.466 | MVPA_w |
| Prevotella oris | Bacteroidota | 95.3 | 2.037 | 0.0023 | 0.0008 | 0.0054 | 0.246 | MVPA_w |
| Prevotella sp000433175 | Bacteroidota | 58.5 | 0.239 | 0.0052 | 0.0016 | 8.50E-04 | 0.188 | MVPA_w |
| CAG.110 sp000435995 | Firmicutes | 40.7 | -3.903 | -0.0047 | 0.0019 | 0.0154 | 0.334 | MVPA_w |
| CAG.273 MGYG.HGUT.04477 | Firmicutes | 59.3 | 0.305 | 0.0047 | 0.0016 | 0.0027 | 0.212 | MVPA_w |
| CAG.95 sp000436115 | Firmicutes | 93.3 | 1.664 | 0.0022 | 0.0009 | 0.0099 | 0.291 | MVPA_w |
| MGYG.HGUT.00340 | Firmicutes | 82.2 | 1.752 | 0.0037 | 0.0014 | 0.0088 | 0.267 | MVPA_w |
| MGYG.HGUT.00456 | Firmicutes | 76.7 | 1.015 | -0.003 | 0.0012 | 0.0153 | 0.334 | MVPA_w |
| MGYG.HGUT.00500 | Firmicutes | 66.3 | 0.391 | -0.0023 | 0.0013 | 0.0745 | 0.468 | MVPA_w |
| MGYG.HGUT.00922 | Firmicutes | 48.9 | -3.070 | -0.0047 | 0.0016 | 0.0038 | 0.234 | MVPA_w |
| MGYG.HGUT.02709 | Firmicutes | 60.7 | 0.316 | -0.0029 | 0.0015 | 0.0512 | 0.466 | MVPA_w |
| MGYG.HGUT.02719 | Firmicutes | 95.5 | 1.953 | -0.0017 | 0.0007 | 0.0129 | 0.321 | MVPA_w |
| MGYG.HGUT.03211 | Firmicutes | 71.6 | 1.772 | -0.0041 | 0.0016 | 0.0111 | 0.304 | MVPA_w |
| MGYG.HGUT.04153 | Firmicutes | 45.8 | -3.686 | -0.0039 | 0.0018 | 0.0278 | 0.401 | MVPA_w |
| MGYG.HGUT.04154 | Firmicutes | 28.6 | -4.406 | -0.0038 | 0.0014 | 0.0059 | 0.246 | MVPA_w |
| Neobitarella massiliensis | Firmicutes | 99.4 | 4.516 | 0.0022 | 0.0007 | 0.0033 | 0.212 | MVPA_w |
| Ruthenibacterium MGYG.HGUT.02155 | Firmicutes | 61.3 | 0.528 | -0.0052 | 0.0018 | 0.0052 | 0.246 | MVPA_w |
| Streptococcus MGYG.HGUT.03136 | Firmicutes | 83.6 | 1.791 | 0.0044 | 0.0013 | 0.0012 | 0.188 | MVPA_w |
| Streptococcus MGYG.HGUT.04266 | Firmicutes | 71.6 | 1.052 | 0.0049 | 0.0015 | 0.0017 | 0.209 | MVPA_w |
| UBA5416 MGYG.HGUT.03192 | Firmicutes | 99.8 | 2.823 | 0.0013 | 0.0005 | 0.0104 | 0.299 | MVPA_w |
| UBA9502 MGYG.HGUT.02280 | Firmicutes | 80.2 | 1.120 | 0.0011 | 0.0013 | 0.4025 | 0.772 | MVPA_w |
| MGYG.HGUT.02643 | Proteobacteria | 27.6 | -4.494 | 0.0034 | 0.0014 | 0.0196 | 0.359 | MVPA_w |
| Collinsella MGYG.HGUT.03062 | Actinobacteriota | 74.2 | 2.533 | 1.1136 | 0.3295 | 7.87E-04 | 0.083 | MVPA_wC |
| Rothia dentocariosa | Actinobacteriota | 26.6 | -4.625 | 0.8986 | 0.2675 | 8.47E-04 | 0.083 | MVPA_wC |
| Bacteroides barnesiae | Bacteroidota | 93.9 | 3.183 | 0.6901 | 0.2159 | 0.0015 | 0.105 | MVPA_wC |
| Bacteroides clarus | Bacteroidota | 95.9 | 3.023 | 0.6703 | 0.2002 | 8.78E-04 | 0.083 | MVPA_wC |
| Bacteroides massiliensis | Bacteroidota | 99.0 | 5.721 | 0.6786 | 0.1876 | 3.29E-04 | 0.078 | MVPA_wC |
| Bacteroides MGYG.HGUT.02568 | Bacteroidota | 44.0 | -3.894 | 0.9981 | 0.2965 | 8.26E-04 | 0.083 | MVPA_wC |
| Bacteroides MGYG.HGUT.04094 | Bacteroidota | 83.0 | 1.527 | 1.0691 | 0.2505 | 2.39E-05 | 0.045 | MVPA_wC |
| Bacteroides MGYG.HGUT.04188 | Bacteroidota | 90.2 | 1.852 | 0.8112 | 0.2192 | 2.41E-04 | 0.078 | MVPA_wC |
| Bacteroides stercoris | Bacteroidota | 99.4 | 5.544 | 0.8862 | 0.2297 | 1.30E-04 | 0.078 | MVPA_wC |
| K10 sp001941205 | Bacteroidota | 22.9 | -4.651 | 0.7105 | 0.2507 | 0.0048 | 0.166 | MVPA_wC |
| MGYG.HGUT.02575 | Bacteroidota | 86.1 | 1.937 | 0.9675 | 0.2668 | 3.19E-04 | 0.078 | MVPA_wC |
| Prevotella oris | Bacteroidota | 95.3 | 2.037 | 0.435 | 0.1534 | 0.0048 | 0.166 | MVPA_wC |
| Prevotella sp000433175 | Bacteroidota | 58.5 | 0.239 | 0.9722 | 0.2938 | 0.001 | 0.085 | MVPA_wC |
| CAG.110 sp000435995 | Firmicutes | 40.7 | -3.903 | -0.618 | 0.3642 | 0.0904 | 0.453 | MVPA_wC |
| CAG.273 MGYG.HGUT.04477 | Firmicutes | 59.3 | 0.305 | 1.1082 | 0.2945 | 1.90E-04 | 0.078 | MVPA_wC |
| CAG.95 sp000436115 | Firmicutes | 93.3 | 1.664 | 0.5499 | 0.1602 | 6.52E-04 | 0.082 | MVPA_wC |
| MGYG.HGUT.00340 | Firmicutes | 82.2 | 1.752 | 0.8916 | 0.2623 | 7.35E-04 | 0.083 | MVPA_wC |
| MGYG.HGUT.00456 | Firmicutes | 76.7 | 1.015 | -0.7007 | 0.2307 | 0.0025 | 0.129 | MVPA_wC |
| MGYG.HGUT.00500 | Firmicutes | 66.3 | 0.391 | -0.8495 | 0.2453 | 5.82E-04 | 0.078 | MVPA_wC |
| MGYG.HGUT.00922 | Firmicutes | 48.9 | -3.070 | -1.0031 | 0.3024 | 9.82E-04 | 0.085 | MVPA_wC |
| MGYG.HGUT.02709 | Firmicutes | 60.7 | 0.316 | -0.886 | 0.2749 | 0.0014 | 0.105 | MVPA_wC |
| MGYG.HGUT.02719 | Firmicutes | 95.5 | 1.953 | -0.4622 | 0.1304 | 4.33E-04 | 0.078 | MVPA_wC |
| MGYG.HGUT.03211 | Firmicutes | 71.6 | 1.772 | -0.9052 | 0.3014 | 0.0028 | 0.135 | MVPA_wC |
| MGYG.HGUT.04153 | Firmicutes | 45.8 | -3.686 | -1.1038 | 0.3343 | 0.001 | 0.085 | MVPA_wC |
| MGYG.HGUT.04154 | Firmicutes | 28.6 | -4.406 | -0.9134 | 0.2611 | 5.14E-04 | 0.078 | MVPA_wC |
| Neobitarella massiliensis | Firmicutes | 99.4 | 4.516 | 0.4922 | 0.1394 | 4.55E-04 | 0.078 | MVPA_wC |
| Ruthenibacterium MGYG.HGUT.02155 | Firmicutes | 61.3 | 0.528 | -0.9036 | 0.3508 | 0.0103 | 0.226 | MVPA_wC |
| Streptococcus MGYG.HGUT.03136 | Firmicutes | 83.6 | 1.791 | 0.9105 | 0.2547 | 3.87E-04 | 0.078 | MVPA_wC |
| Streptococcus MGYG.HGUT.04266 | Firmicutes | 71.6 | 1.052 | 1.0704 | 0.2908 | 2.60E-04 | 0.078 | MVPA_wC |
| UBA5416 MGYG.HGUT.03192 | Firmicutes | 99.8 | 2.823 | 0.3408 | 0.0953 | 3.84E-04 | 0.078 | MVPA_wC |
| UBA9502 MGYG.HGUT.02280 | Firmicutes | 80.2 | 1.120 | 0.8205 | 0.2366 | 5.74E-04 | 0.078 | MVPA_wC |
| MGYG.HGUT.02643 | Proteobacteria | 27.6 | -4.494 | 0.7145 | 0.2718 | 0.0089 | 0.212 | MVPA_wC |
| Collinsella MGYG.HGUT.03062 | Actinobacteriota | 74.2 | 2.533 | 0.0263 | 0.0355 | 0.4591 | 0.998 | TotalSit_hrs |
| Rothia dentocariosa | Actinobacteriota | 26.6 | -4.625 | -0.0042 | 0.0287 | 0.8833 | 0.998 | TotalSit_hrs |
| Bacteroides barnesiae | Bacteroidota | 93.9 | 3.183 | -0.0116 | 0.023 | 0.6153 | 0.998 | TotalSit_hrs |
| Bacteroides clarus | Bacteroidota | 95.9 | 3.023 | -0.0045 | 0.0215 | 0.8345 | 0.998 | TotalSit_hrs |
| Bacteroides massiliensis | Bacteroidota | 99.0 | 5.721 | -0.0133 | 0.02 | 0.5067 | 0.998 | TotalSit_hrs |
| Bacteroides MGYG.HGUT.02568 | Bacteroidota | 44.0 | -3.894 | -0.0486 | 0.0317 | 0.1264 | 0.998 | TotalSit_hrs |
| Bacteroides MGYG.HGUT.04094 | Bacteroidota | 83.0 | 1.527 | -0.0066 | 0.027 | 0.8081 | 0.998 | TotalSit_hrs |
| Bacteroides MGYG.HGUT.04188 | Bacteroidota | 90.2 | 1.852 | -0.0117 | 0.0235 | 0.6174 | 0.998 | TotalSit_hrs |
| Bacteroides stercoris | Bacteroidota | 99.4 | 5.544 | -0.0195 | 0.0248 | 0.4311 | 0.998 | TotalSit_hrs |
| K10 sp001941205 | Bacteroidota | 22.9 | -4.651 | 0.0293 | 0.0267 | 0.272 | 0.998 | TotalSit_hrs |
| MGYG.HGUT.02575 | Bacteroidota | 86.1 | 1.937 | 0.0416 | 0.0288 | 0.1487 | 0.998 | TotalSit_hrs |
| Prevotella oris | Bacteroidota | 95.3 | 2.037 | -0.0073 | 0.0164 | 0.6566 | 0.998 | TotalSit_hrs |
| Prevotella sp000433175 | Bacteroidota | 58.5 | 0.239 | 0.0174 | 0.0313 | 0.5787 | 0.998 | TotalSit_hrs |
| CAG.110 sp000435995 | Firmicutes | 40.7 | -3.903 | 0.0374 | 0.0388 | 0.3361 | 0.998 | TotalSit_hrs |
| CAG.273 MGYG.HGUT.04477 | Firmicutes | 59.3 | 0.305 | 0.0174 | 0.0316 | 0.5813 | 0.998 | TotalSit_hrs |
| CAG.95 sp000436115 | Firmicutes | 93.3 | 1.664 | -0.0011 | 0.0172 | 0.9492 | 0.998 | TotalSit_hrs |
| MGYG.HGUT.00340 | Firmicutes | 82.2 | 1.752 | -0.0095 | 0.0281 | 0.7347 | 0.998 | TotalSit_hrs |
| MGYG.HGUT.00456 | Firmicutes | 76.7 | 1.015 | -0.0514 | 0.0247 | 0.0381 | 0.998 | TotalSit_hrs |
| MGYG.HGUT.00500 | Firmicutes | 66.3 | 0.391 | -0.0254 | 0.0264 | 0.3363 | 0.998 | TotalSit_hrs |
| MGYG.HGUT.00922 | Firmicutes | 48.9 | -3.070 | 0.0045 | 0.0323 | 0.8889 | 0.998 | TotalSit_hrs |
| MGYG.HGUT.02709 | Firmicutes | 60.7 | 0.316 | -0.0054 | 0.0295 | 0.8545 | 0.998 | TotalSit_hrs |
| MGYG.HGUT.02719 | Firmicutes | 95.5 | 1.953 | 0.0048 | 0.014 | 0.7301 | 0.998 | TotalSit_hrs |
| MGYG.HGUT.03211 | Firmicutes | 71.6 | 1.772 | -0.0032 | 0.0322 | 0.9205 | 0.998 | TotalSit_hrs |
| MGYG.HGUT.04153 | Firmicutes | 45.8 | -3.686 | 0.0251 | 0.0359 | 0.4846 | 0.998 | TotalSit_hrs |
| MGYG.HGUT.04154 | Firmicutes | 28.6 | -4.406 | -0.0024 | 0.028 | 0.9322 | 0.998 | TotalSit_hrs |
| Neobitarella massiliensis | Firmicutes | 99.4 | 4.516 | -0.0055 | 0.0149 | 0.7102 | 0.998 | TotalSit_hrs |
| Ruthenibacterium MGYG.HGUT.02155 | Firmicutes | 61.3 | 0.528 | 0.051 | 0.0372 | 0.1718 | 0.998 | TotalSit_hrs |
| Streptococcus MGYG.HGUT.03136 | Firmicutes | 83.6 | 1.791 | -0.0058 | 0.0272 | 0.8308 | 0.998 | TotalSit_hrs |
| Streptococcus MGYG.HGUT.04266 | Firmicutes | 71.6 | 1.052 | -0.0042 | 0.0311 | 0.8918 | 0.998 | TotalSit_hrs |
| UBA5416 MGYG.HGUT.03192 | Firmicutes | 99.8 | 2.823 | -0.006 | 0.0103 | 0.5585 | 0.998 | TotalSit_hrs |
| UBA9502 MGYG.HGUT.02280 | Firmicutes | 80.2 | 1.120 | -0.0116 | 0.0256 | 0.651 | 0.998 | TotalSit_hrs |
| MGYG.HGUT.02643 | Proteobacteria | 27.6 | -4.494 | 0.0303 | 0.0291 | 0.2984 | 0.998 | TotalSit_hrs |
| ^a^ Centered log-ratio transformed abundance of taxa with a prevalence of >20% were tested for association with daily sitting hours (TotalSit_hrs) and moderate to vigorous physical activity (MVPA) measures, including continuous MVPA related to exercise/sport (MVPA_s), work/home (MVPA_w), and total (MVPA_a) as well as categorical MVPA measures (MVPA_sC, MVPA_wC, and MVPA_aC). The general linear regression model was adjusted for enrollment age, sex, time interval between enrollment and stool collection, body mass index, education, household income, tobacco smoking, alcohol drinking, total energy intake, healthy eating index, daily sitting hours, and history of selected chronic diseases at baseline including cancer, CVD, diabetes, and hypertension. | | | | | | | | |

| **Supplemental Table 3**. Microbial MetaCyC pathways associated with physical activity measures in all selected SCCS participants | | | | | | | |
| --- | --- | --- | --- | --- | --- | --- | --- |
| Metabolic pathways | Prevalence | Median Arcsine square root transformed relative abundance | Beta^a^ | Se^a^ | *P*^a^ | Fdr^a^ | PA measure |
| All-trans-farnesol biosynthesis | 99.2 | 0.0589 | 3.06E-05 | 8.74E-06 | 5.01E-04 | 0.054 | MVPA_a |
| Cis-vaccenate biosynthesis | 100 | 0.1329 | 3.41E-05 | 9.54E-06 | 3.87E-04 | 0.054 | MVPA_a |
| dTDP-&beta;D-fucofuranose biosynthesis | 58.1 | 0.0095 | 2.03E-05 | 1.20E-05 | 0.0928 | 0.620 | MVPA_a |
| L-ascorbate biosynthesis IV | 22.1 | 0 | 1.79E-05 | 5.34E-06 | 8.43E-04 | 0.072 | MVPA_a |
| L-aspartate and L-asparagine biosynthesis | 100 | 0.1306 | 2.33E-05 | 5.77E-06 | 6.40E-05 | 0.027 | MVPA_a |
| L-citrulline biosynthesis | 100 | 0.103 | 3.30E-05 | 1.19E-05 | 0.0059 | 0.229 | MVPA_a |
| Taxadiene biosynthesis engineered | 99.2 | 0.0837 | 3.99E-05 | 1.13E-05 | 4.49E-04 | 0.054 | MVPA_a |
| Urea cycle | 100 | 0.0928 | 2.99E-05 | 1.27E-05 | 0.0185 | 0.298 | MVPA_a |
| Xyloglucan degradation II (exoglucanase) | 57.7 | 0.007 | 1.47E-05 | 1.06E-05 | 0.1655 | 0.713 | MVPA_a |
| All-trans-farnesol biosynthesis | 99.2 | 0.0589 | 0.005 | 0.002 | 0.0028 | 0.160 | MVPA_aC |
| Cis-vaccenate biosynthesis | 100 | 0.1329 | 0.005 | 0.002 | 0.0083 | 0.169 | MVPA_aC |
| dTDP-&beta;D-fucofuranose biosynthesis | 58.1 | 0.0095 | 0.009 | 0.002 | 7.96E-05 | 0.033 | MVPA_aC |
| L-ascorbate biosynthesis IV | 22.1 | 0 | 2.49E-04 | 0.001 | 0.8129 | 0.997 | MVPA_aC |
| L-aspartate and L-asparagine biosynthesis | 100 | 0.1306 | 0.004 | 0.001 | 2.44E-04 | 0.033 | MVPA_aC |
| L-citrulline biosynthesis | 100 | 0.103 | 0.008 | 0.002 | 0.0011 | 0.091 | MVPA_aC |
| Taxadiene biosynthesis engineered | 99.2 | 0.0837 | 0.007 | 0.002 | 0.003 | 0.160 | MVPA_aC |
| Urea cycle | 100 | 0.0928 | 0.009 | 0.002 | 3.06E-04 | 0.033 | MVPA_aC |
| Xyloglucan degradation II (exoglucanase) | 57.7 | 0.007 | 0.007 | 0.002 | 2.75E-04 | 0.033 | MVPA_aC |
| All-trans-farnesol biosynthesis | 99.2 | 0.0589 | 4.74E-05 | 5.59E-05 | 0.3966 | 0.996 | MVPA_s |
| Cis-vaccenate biosynthesis | 100 | 0.1329 | 7.80E-05 | 6.10E-05 | 0.2012 | 0.996 | MVPA_s |
| dTDP-&beta;D-fucofuranose biosynthesis | 58.1 | 0.0095 | 2.58E-05 | 7.62E-05 | 0.7344 | 0.996 | MVPA_s |
| L-ascorbate biosynthesis IV | 22.1 | 0 | 3.75E-05 | 3.41E-05 | 0.2711 | 0.996 | MVPA_s |
| L-aspartate and L-asparagine biosynthesis | 100 | 0.1306 | 8.59E-05 | 3.69E-05 | 0.0201 | 0.996 | MVPA_s |
| L-citrulline biosynthesis | 100 | 0.103 | 6.07E-05 | 7.60E-05 | 0.4247 | 0.996 | MVPA_s |
| Taxadiene biosynthesis engineered | 99.2 | 0.0837 | 7.54E-05 | 7.22E-05 | 0.2969 | 0.996 | MVPA_s |
| Urea cycle | 100 | 0.0928 | 5.70E-05 | 8.04E-05 | 0.4788 | 0.996 | MVPA_s |
| Xyloglucan degradation II (exoglucanase) | 57.7 | 0.007 | 6.76E-06 | 6.70E-05 | 0.9196 | 0.996 | MVPA_s |
| All-trans-farnesol biosynthesis | 99.2 | 0.0589 | 0.002 | 0.002 | 0.4236 | 0.954 | MVPA_sC |
| Cis-vaccenate biosynthesis | 100 | 0.1329 | 0.001 | 0.002 | 0.51 | 0.954 | MVPA_sC |
| dTDP-&beta;D-fucofuranose biosynthesis | 58.1 | 0.0095 | 0.001 | 0.003 | 0.6931 | 0.964 | MVPA_sC |
| L-ascorbate biosynthesis IV | 22.1 | 0 | -6.94E-05 | 0.0011713 | 0.9528 | 0.996 | MVPA_sC |
| L-aspartate and L-asparagine biosynthesis | 100 | 0.1306 | 0.003 | 0.001 | 0.0186 | 0.734 | MVPA_sC |
| L-citrulline biosynthesis | 100 | 0.103 | 0.001 | 0.003 | 0.5727 | 0.954 | MVPA_sC |
| Taxadiene biosynthesis engineered | 99.2 | 0.0837 | 0.001 | 0.002 | 0.5617 | 0.954 | MVPA_sC |
| Urea cycle | 100 | 0.0928 | 1.20E-04 | 2.76E-03 | 0.9653 | 0.997 | MVPA_sC |
| Xyloglucan degradation II (exoglucanase) | 57.7 | 0.007 | 0.002 | 0.002 | 0.4413 | 0.954 | MVPA_sC |
| All-trans-farnesol biosynthesis | 99.2 | 0.0589 | 3.18E-05 | 9.09E-06 | 5.06E-04 | 0.055 | MVPA_w |
| Cis-vaccenate biosynthesis | 100 | 0.1329 | 3.48E-05 | 9.92E-06 | 5.07E-04 | 0.055 | MVPA_w |
| dTDP-&beta;D-fucofuranose biosynthesis | 58.1 | 0.0095 | 2.12E-05 | 1.25E-05 | 0.0907 | 0.622 | MVPA_w |
| L-ascorbate biosynthesis IV | 22.1 | 0 | 1.84E-05 | 5.55E-06 | 0.001 | 0.086 | MVPA_w |
| L-aspartate and L-asparagine biosynthesis | 100 | 0.1306 | 2.28E-05 | 6.01E-06 | 1.65E-04 | 0.055 | MVPA_w |
| L-citrulline biosynthesis | 100 | 0.103 | 3.41E-05 | 1.24E-05 | 0.0063 | 0.256 | MVPA_w |
| Taxadiene biosynthesis engineered | 99.2 | 0.0837 | 4.11E-05 | 1.18E-05 | 5.11E-04 | 0.055 | MVPA_w |
| Urea cycle | 100 | 0.0928 | 3.08E-05 | 1.32E-05 | 0.0197 | 0.307 | MVPA_w |
| Xyloglucan degradation II (exoglucanase) | 57.7 | 0.007 | 1.57E-05 | 1.10E-05 | 0.1541 | 0.719 | MVPA_w |
| All-trans-farnesol biosynthesis | 99.2 | 0.0589 | 0.006 | 0.002 | 0.0012 | 0.099 | MVPA_wC |
| Cis-vaccenate biosynthesis | 100 | 0.1329 | 0.005 | 0.002 | 0.0168 | 0.290 | MVPA_wC |
| dTDP-&beta;D-fucofuranose biosynthesis | 58.1 | 0.0095 | 0.011 | 0.002 | 3.53E-06 | 0.002 | MVPA_wC |
| L-ascorbate biosynthesis IV | 22.1 | 0 | -9.45E-05 | 0.0010637 | 0.9292 | 0.971 | MVPA_wC |
| L-aspartate and L-asparagine biosynthesis | 100 | 0.1306 | 0.003 | 0.001 | 0.004 | 0.143 | MVPA_wC |
| L-citrulline biosynthesis | 100 | 0.103 | 0.008 | 0.002 | 8.11E-04 | 0.099 | MVPA_wC |
| Taxadiene biosynthesis engineered | 99.2 | 0.0837 | 0.007 | 0.002 | 0.002 | 0.123 | MVPA_wC |
| Urea cycle | 100 | 0.0928 | 0.009 | 0.002 | 4.33E-04 | 0.093 | MVPA_wC |
| Xyloglucan degradation II (exoglucanase) | 57.7 | 0.007 | 0.007 | 0.002 | 0.001 | 0.099 | MVPA_wC |
| All-trans-farnesol biosynthesis | 99.2 | 0.0589 | -0.000277827 | 1.83E-04 | 0.1304 | 0.510 | TotalSit_hrs |
| Cis-vaccenate biosynthesis | 100 | 0.1329 | -1.27E-05 | 2.01E-04 | 0.9495 | 0.990 | TotalSit_hrs |
| dTDP-&beta;D-fucofuranose biosynthesis | 58.1 | 0.0095 | -0.000194573 | 2.53E-04 | 0.4415 | 0.688 | TotalSit_hrs |
| L-ascorbate biosynthesis IV | 22.1 | 0 | -4.94E-05 | 1.12E-04 | 0.6598 | 0.833 | TotalSit_hrs |
| L-aspartate and L-asparagine biosynthesis | 100 | 0.1306 | -3.89E-05 | 1.21E-04 | 0.7483 | 0.902 | TotalSit_hrs |
| L-citrulline biosynthesis | 100 | 0.103 | 1.13E-04 | 2.51E-04 | 0.653 | 0.832 | TotalSit_hrs |
| Taxadiene biosynthesis engineered | 99.2 | 0.0837 | -0.000499017 | 2.37E-04 | 0.0359 | 0.484 | TotalSit_hrs |
| Urea cycle | 100 | 0.0928 | -7.31E-06 | 2.66E-04 | 0.9781 | 0.992 | TotalSit_hrs |
| Xyloglucan degradation II (exoglucanase) | 57.7 | 0.007 | 1.52E-04 | 2.22E-04 | 0.4945 | 0.730 | TotalSit_hrs |
| ^a^ Arcsine square root transformed relative abundance of pathways with a prevalence of >20% were tested for association with daily sitting hours (TotalSit_hrs) and moderate to vigorous physical activity (MVPA) measures, including continuous MVPA related to exercise/sport (MVPA_s), work/home (MVPA_w), and total (MVPA_a) as well as categorical MVPA measures (MVPA_sC, MVPA_wC, and MVPA_aC). The general linear regression model was adjusted for enrollment age, sex, time interval between enrollment and stool collection, body mass index, education, household income, tobacco smoking, alcohol drinking, total energy intake, healthy eating index, daily sitting hours, and history of selected chronic diseases at baseline including cancer, CVD, diabetes, and hypertension. | | | | | | | |

| **Supplemental Table 4.** Significant interactions in physical activity measures and gut microbial species associations | | | | | | | |
| --- | --- | --- | --- | --- | --- | --- | --- |
| Physical activity | Species | Beta (se)^a^ | *p*^a^ |  | Beta (se)^a^ | *p*^a^ | *p* for interaction^b^ |
|  |  | Ever smokers (n=93) | |  | Never smokers (n=396) | |  |
| MVPA_aC | *MGYG.HGUT.00922* | -1.814 (0.412) | 1.64E-05 |  | 0.321 (0.445) | 0.4714 | 0.0003 |
| MVPA_sC | *CAG.95 sp000436115* | 0.634 (0.265) | 0.0177 |  | -0.439 (0.241) | 0.0698 | 0.0009 |
| ^a^ Centered log‐ratio transformed counts of each species was regressed on PA measures, adjusted for enrollment age, sex, time interval between enrollment and stool collection, body mass index, education, household income, tobacco smoking (not for stratified analysis by smoking status), alcohol drinking, total energy intake, healthy eating index, daily sitting hours, and history of selected chronic diseases at baseline including cancer, cardiovascular disease, diabetes, and hypertension. | | | | | | | |
| ^b^ False discovery rate < 0.10. | | | | | | | |

| **Supplemental Table 5.** Significant interactions in physical activity measures and metabolic pathway associations | | | | | | | |
| --- | --- | --- | --- | --- | --- | --- | --- |
| Physical activity | Pathway | Beta (se)^a^ | *P*^a^ |  | Beta (se)^a^ | *P*^a^ | *p* for interaction^b^ |
|  |  | **Annual household income** | | | | |  |
|  |  | **<$15,000 (n=207)** | |  | **>$15,000l (n=282)** | |  |
| MVPA_s | L-ascorbate biosynthesis IV | 8.49E-5 (4.84E-5) | 0.0811 |  | -7.46E-5 (4.83E-5) | 0.1234 | 0.0067 |
| MVPA_sC | L-ascorbate biosynthesis IV | 0.004 (0.002) | 0.0648 |  | -0.004 (0.001) | 0.0041 | 0.0001 |
| ^a^  Arcsine square root transformed relative abundance of pathways was regressed on PA measures, adjusted for enrollment age, sex, time interval between enrollment and stool collection, body mass index, education, household income (not for stratification analysis), tobacco smoking, alcohol drinking, total energy intake, healthy eating index, daily sitting hours, and history of selected chronic diseases at baseline including cancer, cardiovascular disease, diabetes, and hypertension. | | | | | | | |
| ^b^ False discovery rate < 0.10. | | | | | | | |
